# Machine Learning Classification of Bipolar Disorder Across Mood States Using Natural Language Processing and Speech Analysis: Observational Study

**DOI:** 10.2196/98094

**Published:** 2026-11-01

**Authors:** Yusei Yamaguchi, Yoshinobu Kano, Genichi Sugihara, Ryo Takemura, Hironobu Nakamura, Masaaki Shimizu, Mari Iizuka, Momoko Kitazawa, Ayako Sento, Hidehiko Takahashi, Taishiro Kishimoto

**Affiliations:** 1 Department of Psychiatry and Behavioral Sciences, Graduate School of Medical and Dental Sciences, Institute of Science Tokyo, 1-5-45 Yushima, Bunkyo-ku, Tokyo, Japan; 2 Faculty of Informatics, Shizuoka University, Chuo-ku, Hamamatsu, Shizuoka, Japan; 3 Clinical and Translational Research Center, Keio University Hospital, Shinjuku-ku, Tokyo, Japan; 4 Department of Neuropsychiatry, Keio University School of Medicine, Shinjuku-ku, Tokyo, Japan; 5 Center for the Promotion of Interdisciplinary Research in Medicine and Life Science, School of Medicine, Keio University, 35 Shinanomachi, Shinjuku-ku, Tokyo, 160-8582, Japan, 81 3-5360-1524; 6 Center for Brain Integration Research, Institute of Science Tokyo, Tokyo, Japan

**Keywords:** bipolar disorder, speech analysis, natural language processing, machine learning, acoustic biomarkers, mood states, LASSO, digital health, mel-frequency cepstral coefficients, permutation testing, least absolute shrinkage and selection operator

## Abstract

**Background:**

Bipolar disorder (BP) affects 1% to 3% of adults worldwide, with diagnostic delays averaging 6 years and high initial misdiagnosis rates. The absence of objective diagnostic measures contributes to clinical uncertainty and suboptimal outcomes.

**Objective:**

This study aimed to (1) develop a machine learning model for BP diagnosis and mood state classification using linguistic and acoustic features, (2) identify features associated with different mood states by comparing each with healthy controls (HC), and (3) explore whether features distinguish between mood states within BP.

**Methods:**

We analyzed data prospectively collected over 7 years from 61 patients with BP and 99 HC across Japan. Speech samples were collected via semistructured interviews over 1 to 5 visits and analyzed cross-sectionally, using aggregated or single-visit observations per participant rather than within-person longitudinal modeling. We extracted 6373 acoustic features (openSMILE 3.0, ComParE 2016) and 211 linguistic features. Least absolute shrinkage and selection operator (LASSO)–regularized logistic regression with nested cross-validation and age- and sex-matched undersampling was used. Mood states were classified as depressive state (DS; Hamilton Depression Rating Scale-17 [HAMD-17] ≥8, Young Mania Rating Scale [YMRS] ≤7), state with manic/mixed symptoms (MS/MX; YMRS ≥8), or euthymic state (ES; HAMD-17 ≤7, YMRS ≤7). Seven classification tasks included BP vs HC, each mood state vs HC, and interstate comparisons. Feature importance was validated via permutation testing (*P*<.05). Medication sensitivity analyses were performed.

**Results:**

The model achieved high discrimination for BP vs HC (area under the receiver operating characteristic curve [AUC]=0.916, 95% CI 0.854‐0.958; sensitivity 0.933; specificity 0.859) and strong performance for mood states vs HC (AUC=0.805‐0.874). Acoustic features comprised 96% to 98% of the features achieving statistical significance (*P*<.05), including mel-frequency cepstral coefficients, spectral characteristics, and voice quality measures, showing strong correlations with diagnostic probabilities (|ρ|=0.35‐0.85) and symptom severity (HAMD-17: ρ=–0.51; YMRS: ρ=–0.37); 2 to 4 linguistic features per comparison also reached significance. With sample independence ensured for interstate comparisons, DS vs ES significantly exceeded chance (AUC=0.760, permutation *P*=.03), whereas MS/MX vs ES (AUC=0.601, *P*=.20) and DS vs MS/MX (AUC=0.569, *P*=.30) did not, suggesting that markers primarily reflect persistent disease characteristics with partial state-associated components in depressive states.

**Conclusions:**

The combined acoustic-linguistic model, driven primarily by acoustic features, shows potential as an objective aid for BP diagnosis (AUC>0.9), largely free of medication confounding; some features remained detectable during euthymia, although ES discrimination could not be fully separated from concurrent antipsychotic treatment. Although DS showed partial distinguishability from ES, overall limitations remain for monitoring mood state changes. Findings require validation in diverse populations and against other psychiatric conditions.

## Introduction

Bipolar disorder (BP) is a severe psychiatric condition affecting 1% to 3% of adults worldwide [1], significantly impairing psychosocial functioning, including work performance, financial management, cognitive abilities, and interpersonal relationships [2]. BP is associated with a heightened risk of suicide, with lifetime attempt rates around 20% and mortality rates between 10% and 20% [3-5]. Initial diagnosis presents considerable challenges: at first presentation, 76.8% of patients are misdiagnosed with unipolar depression [6], leading to an average diagnostic delay of approximately 6 years [7]. This delay often results in inappropriate treatment, such as antidepressant monotherapy, which may induce manic or mixed episodes [8]. Accurate mood state assessment is critical for selecting appropriate treatment, as pharmacological strategies differ markedly between depressive and manic or hypomanic phases [9-11].

These diagnostic challenges underscore the need for objective measures. Recent advances in computational methods, particularly natural language processing (NLP) and speech analysis, show promise in detecting linguistic and vocal patterns that may serve as biomarkers for BP. NLP enables quantitative analysis of linguistic patterns reflecting mood states, including use of negative expressions, higher frequency of first-person singular pronouns, and reduced lexical diversity and semantic coherence in individuals with BP [12-14]. Speech analysis of acoustic patterns has identified distinct vocal changes across mood states. Depressive episodes are characterized by flattened intonation, slower speech rate, and reduced vocal intensity [15]. In contrast, manic episodes typically feature rapid speech (pressure of speech) and elevated vocal volume, possibly related to hyperarousal [16]. Furthermore, mel-frequency cepstral coefficients (MFCCs), which are commonly used in speech processing to represent the spectral properties of voice signals, have been linked to symptom severity in unipolar depressive disorder [17-19], and their utility as a state marker in BP has also been proposed [20].

Despite these promising findings, current research on digital biomarkers faces several limitations. Most studies rely on small samples and controlled settings, resulting in inconsistent outcomes. A recent meta-analysis of machine learning applications in BP diagnosis reported a pooled classification accuracy of 0.77 (95% CI 0.75‐0.80), with performance declining as sample size increased [21]. Although some recent studies have explored multimodal approaches [22,23], most analyze NLP and speech features independently, leaving it unclear whether linguistic content or acoustic characteristics offer better diagnostic insight. Multimodal approaches that integrate both linguistic and acoustic features may provide complementary information, potentially improving diagnostic accuracy by capturing both content-related and delivery-related aspects of speech. Moreover, few studies have jointly analyzed NLP and acoustic features across different mood states in BP.

This 7-year (2017‐2024) prospective observational study involving 61 patients with BP and 99 healthy control (HC) individuals aimed to achieve three main objectives: (1) to enhance diagnostic accuracy through combined analysis of NLP and speech features, (2) to identify acoustic and linguistic features associated with different mood states (depressive state, state with manic/mixed symptoms, and euthymic state) by comparing each state with HC, and (3) to explore whether the identified features distinguish between different mood states within BP. This research contributes to understanding the linguistic and acoustic characteristics of BP across mood states, with potential implications for developing objective diagnostic tools.

## Methods

### Participants

This study was conducted as part of the Understanding Psychiatric Illness through Natural Language Processing (UNDERPIN) project [24]. It included 61 patients with BP and 99 HC participants from the UNDERPIN dataset. Patients were recruited from the outpatient and inpatient services of Keio University Hospital and collaborating institutions (7 hospitals and 3 outpatient clinics across Japan) through referral by their treating psychiatrists. Inclusion criteria for patients were as follows: age ≥20 years, a diagnosis of BP per Diagnostic and Statistical Manual of Mental Disorders 5th Edition (DSM-5) [25] or International Classification of Diseases 11th Revision (ICD-11) [26] criteria; and inpatient or outpatient status. Exclusion criteria included comorbid diagnoses of major depressive disorder, schizophrenia, anxiety disorders, or dementia, as defined by DSM-5 or ICD-11 criteria. These conditions were specified as exclusion criteria because the UNDERPIN project concurrently enrolled participants with these diagnoses, and the presence of such comorbidities could confound the characterization of speech and language features specific to BP. Among the 61 patients with BP, 18 were inpatients and 43 were outpatients at the time of recruitment. The HC group consisted of volunteers aged ≥20 years without any history of psychiatric disorders. All participants were fluent in Japanese and provided written informed consent after receiving a full explanation of the study. Data were collected from 2017 to 2024. HC individuals responded to advertisements posted on the study website and in print media and were screened using the Mini-International Neuropsychiatric Interview (MINI) and the Mini-Mental State Examination (MMSE) [27,28]; they were excluded if any psychiatric diagnosis was identified or if the MMSE score was below 27. Throughout the study period, no patient in the BP group had a change in diagnostic category; across the full UNDERPIN source dataset, no participant changed diagnostic category from another disorder to BP or from BP to another disorder. Within-patient variation in mood state (depressive, manic/mixed, and euthymic) was observed and was labeled at each visit, but this reflects fluctuation in clinical state rather than a change in diagnosis.

### Clinical Assessment and Data Collection

Participants completed between 1 and 5 assessment visits to monitor clinical status changes (BP: median 3 [IQR 1-5]; HC: median 2 [IQR 2-2]). Consecutive visits were a median of 51 (IQR 35‐91) days apart for patients with BP and 35 (IQR 28‐49; minimum 18) days for HC, with individual follow-up extending to 37 months. A descriptive summary of the temporal structure of data collection is provided in Multimedia Appendix 1 (section 15). Of the 61 patients with BP, 42 were recorded in a single mood state across all of their visits, whereas 19 were recorded in 2 or more states. Trained psychiatrists or psychologists assessed symptom severity using the Hamilton Depression Rating Scale 17 (HAMD-17) and the Young Mania Rating Scale (YMRS) [29,30]. At each visit, the concurrent diagnosis, current medications, and adverse events were also recorded, and each visit was labeled with the diagnosis and symptom severity assessed at that time point.

Each interview lasted 30 to 60 minutes and included three tasks: (1) free conversation, (2) picture description, and (3) storytelling. Free conversation involved discussions with psychologists or psychiatrists on topics such as illness history, current status, daily life, sleep patterns, hobbies, work, and recent events. These conversations were intentionally unstructured to elicit natural speech patterns. Interviewers adhered to specific guidelines: using neutral words, avoiding emotional terms, making interjections to facilitate dialogue, and following up on the participant’s account.

For picture description, participants described 3 images, that is, children eating donuts from the Visual Perception Test for Agnosia, waterside from the Neurobehavioral Cognitive Status Examination (COGNISTAT), and shore from the Western Aphasia Battery, in as much detail as possible. In the storytelling task, participants narrated Cinderella or the famous Japanese folktale *Kaguya-Hime* based on familiarity. Both tasks took approximately 10 minutes each. Procedures were standardized across participants.

All interviews were recorded using standardized microphone setups in soundproof booths or quiet rooms to minimize ambient noise. Stereo recording was performed using 2 types of audio interface systems manufactured by Roland Corporation and Tascam Corporation. In a 2-channel configuration, each interview used 2 microphones, with the left channel assigned to the interviewer and the right channel assigned to the participant, enabling postrecording separation of participant speech. Each assessment incorporated these 3 tasks, as outlined in our study protocol [24]. Recordings had a sampling rate of 16 kHz and a bit depth of 16 bits. Free conversations accounted for 60% to 80% of each interview [24]. For this analysis, data from all 3 tasks were merged into a unified dataset to capture general speech characteristics rather than task-specific variations.

Medication data were collected at each assessment visit. For patients with BP (median 3 [IQR 1-5] visits, range 1‐5), all medications used during the observation period were recorded. Consistent with our analytical approach of averaging acoustic and linguistic features across visits to create a single data point per participant, a participant was classified as using a medication class if they received at least 1 medication from that class at any visit during the study period. Medications were categorized into 5 classes: antipsychotics, mood stabilizers, antidepressants, anxiolytics, and others (primarily medications for physical comorbidities). Inpatient or outpatient status was determined at the time of recruitment.

### Data Collection and Feature Extraction Overview

We collected speech samples during semistructured interviews across multiple assessments (1‐5 visits) and transcribed them for NLP analysis. Our assessment pipeline integrated comprehensive NLP techniques (including sentiment, morphological, and syntactic analyses, generating 211 linguistic features) with speech analysis using openSMILE 3.0 (audEERING GmbH), a toolkit for speech and emotion recognition, which extracted 6373 acoustic features. Feature selection was performed using machine learning techniques as described in the *Machine Learning Classification* section.

### NLP Analyses

A professional transcription service transcribed participant speech for NLP analyses. Interviewer speech was removed, and only the participant’s utterances were analyzed. Our analysis used 3 NLP techniques to extract 211 linguistic features: (1) sentiment analysis (Plutchik’s 8 primary emotions: joy, sadness, anger, fear, trust, disgust, anticipation, and surprise [31]), (2) morphological analysis (word choice and grammatical patterns), and (3) syntactic analysis (sentence structure, including signs of rapid thinking or flight of ideas). It is important to note that these NLP features quantify linguistic patterns (eg, syntactic complexity, part-of-speech distributions, and emotional expression patterns) rather than directly assessing clinical symptoms. For example, syntactic analysis using GiNZA (Megagon Labs; see Multimedia Appendix 1, section 3) extracts objective measures of sentence structure complexity and dependency relationships, which may hypothetically reflect cognitive processes such as rapid thinking, but these features represent quantifiable linguistic patterns rather than symptom assessments. This distinction is crucial: while clinical observations rely on subjective interpretation of symptoms, our NLP approach provides objective numerical quantification of linguistic patterns that may be associated with mood states. Full methodological details are available in Multimedia Appendix 1 (sections 1‐3).

### Speech Analyses

Audio channels were separated to isolate participant speech, and recording quality was verified by trained personnel to ensure data integrity. Data integrity checks included verification that participant and interviewer speech were correctly separated, assessment of noise contamination, and evaluation of recording quality. We used openSMILE 3.0 software with the ComParE 2016 feature set [32,33], which contains 6373 acoustic features (Multimedia Appendix 1, section 4). This set has been used in diverse paralinguistic contexts [20,34,35] and was selected for its comprehensive coverage of features relevant to mood states. Features heavily influenced by volume, which could not be standardized due to variable microphone-to-mouth distances, were excluded before model training (4 openSMILE feature families: pcm_RMSenergy, audspec_lengthL1norm, audspecRasta_lengthL1norm, and audSpec_Rfilt; 2900 features), leaving 3473 acoustic features as candidate predictors.

Following prior work [20], we organized the ComParE 2016 acoustic features into prosodic (pitch, energy, loudness, speaking rate, and pauses), spectral (spectral energy distribution, including MFCCs), and voice quality (glottal airflow and vocal fold vibration, such as jitter, shimmer, and harmonics-to-noise ratio) features. As noted above, volume-dependent features were excluded. Because MFCCs are among the acoustic markers most consistently reported in depression and BP [17-20], MFCCs were separated from the remaining spectral features and treated as a distinct category. The significant acoustic features are therefore reported in 3 categories: MFCC, spectral, and voice quality.

MFCCs are the coefficients obtained by applying a discrete cosine transform to the logarithm of the output energies of a mel-scaled filter bank and retaining only a small number of low-order coefficients (on the order of 13), which yields a smooth estimate of the spectral envelope reflecting the resonance characteristics of the vocal tract [36,37]. MFCC 0 represents the average power of the spectrum, MFCC 1 approximates the broad shape of the spectrum, and higher-order coefficients represent progressively finer spectral detail of the envelope across frequency [38,39]. In practice, the first 8 to 13 coefficients are used to represent the shape of the spectrum, whereas capturing pitch and tone information can require higher-order coefficients—for example, up to around 20 coefficients in tonal language speech recognition [39]; because the present study retained coefficients only up to MFCC 12, all of them lie within the low-quefrency range that describes the envelope and are therefore interpreted as reflecting the vocal tract shape (timbre) rather than the fundamental frequency [37,39]. Coefficient order and temporal functionals (eg, flatness) describe 2 independent properties of a feature: the former reflects which part of the spectral envelope a coefficient captures, and the latter reflects how that value varies over the course of an utterance.

To illustrate acoustic differences, we used a WORLD vocoder-based approach to manipulate representative speech samples, presenting both the modified waveforms and corresponding feature values (Multimedia Appendix 1, section 5). This method is widely used in voice conversion for its balance and interpretability.

### Machine Learning Classification

The minimum sample size for the primary BP vs HC classification was determined by an a priori power analysis in three steps: (1) discrimination AUCs of 0.71‐0.76, reported by prior speech- and NLP-based BP classification studies [40,41], were adopted as the expected effect magnitude; (2) these AUCs were converted to Cohen *d* using the area-to-*d* conversion of Rice and Harris [42], giving d=0.78 to 1; and (3) this range was entered into an independent 2-sample *t* test power analysis (2-tailed *α*=.05, power=0.8) in G*Power [43], which indicated a requirement of 17 participants per group at d=1 and 27 participants per group at d=0.78. BP vs HC (n=60 vs n=99) and depressive state (DS) vs HC (n=32 vs n=97) met this requirement at both effect sizes, whereas euthymic state (ES) vs HC (n=26 vs n=97) and state with manic or mixed symptoms (MS/MX) vs HC (n=20 vs n=97) met it only at d=1. Among the interstate subgroups (n=15‐27), all but 1 fell below the 27 required at d=0.78, and the smallest subgroup (n=15) also fell below the 17 required at d=1 (see *Limitations*). The YMRS threshold of ≥8 was selected to capture the presence of manic symptoms, including subthreshold manic symptoms (YMRS 8‐12), which have been shown to have clinical significance [44,45]. Due to the limited sample size, states with manic or mixed symptoms were merged for analysis. Among the 20 participants in the MS/MX group, 8 had predominantly manic symptoms and 12 had mixed features.

We performed 7 classification tasks:

Primary HC comparisons:

- BP (n=60) vs HC (n=99)
- DS (HAMD-17 ≥8, YMRS ≤7; n=32) vs HC
- MS/MX (YMRS ≥8; n=20) vs HC
- ES (HAMD-17 ≤7, YMRS ≤7; n=26) vs HC

Direct mood state comparisons:

- DS vs ES
- MS/MX vs ES
- DS vs MS/MX

One data point per participant ensured sample independence. While data were collected prospectively over 7 years (2017‐2024), the analytical approach was cross-sectional: each participant contributed a single aggregated data point to ensure sample independence rather than being analyzed longitudinally across visits. For BP vs HC, data from 1 to 5 visits were averaged to create a single representative point. For mood state vs HC analyses (DS vs HC, MS/MX vs HC, and ES vs HC), 1 qualifying visit was randomly selected. All tasks were combined for each data point. In accordance with the UNDERPIN protocol, each participant was assessed between 2 and 5 times during the study period, with a minimum interval of at least 1 month between consecutive assessments. For direct interstate comparisons (DS vs ES, MS/MX vs ES, DS vs MS/MX), 1 qualifying visit per participant per mood state was randomly selected. To ensure sample independence, participants who met criteria for more than 1 mood state across visits were excluded from both groups in each comparison. This resulted in the following sample sizes: DS vs ES (DS: n=23, ES: n=17), MS/MX vs ES (MS/MX: n=15, ES: n=21; 5 excluded), and MS/MX vs DS (MS/MX: n=15, DS: n=27). Averaging across a participant’s visits provided a stable, occasion-independent estimate for the trait-level BP vs HC comparison; although this collapsed within-participant variation for that comparison, such variation was examined directly in the mood state analyses (DS vs HC, MS/MX vs HC, and ES vs HC), which used the state-specific visit rather than the average. We did not perform within-person longitudinal (trajectory) modeling because the study was designed to sample mood states for cross-sectional discrimination rather than to model temporal change and because the irregular and sparse visit schedule (1-5 visits per participant) together with the small mood state subgroups would render such modeling underpowered; modeling within-person change over time is an important direction for future work.

In this study, we used an operational definition of trait markers. Specifically, we defined “trait markers” as features that were statistically significant in the ES vs HC comparison, where patients were in ES with minimal symptoms (HAMD-17 ≤7, YMRS ≤7). Because these features remained detectable during remission, we interpreted them as disease-associated characteristics that are not primarily driven by acute mood symptoms. We acknowledge that definitive establishment of “trait” status would require longitudinal within-person confirmation, which our cross-sectional analytical design does not provide.

It should be noted that this analysis approach, which averages or selects single visits per participant, does not constitute a longitudinal analysis of within-participant changes over time. Rather, this cross-sectional approach was designed to maintain sample independence while maximizing data use.

Prior to model training, all features were standardized to ensure that features from different modalities (linguistic and acoustic) were on comparable scales, preventing features with larger numerical ranges from dominating the model. We applied least absolute shrinkage and selection operator (LASSO)–regularized logistic regression using acoustic and linguistic features as the input parameters [46]. Because the acoustic and linguistic feature sets differ markedly in dimensionality (3473 vs 211), we additionally performed modality-specific analyses in which the linguistic-only, acoustic-only, and combined feature sets were each submitted to the identical nested cross-validation and LASSO procedure, with model-level significance assessed by a permutation test (100 permutations) in which the diagnostic labels were shuffled at the participant level.

Nested cross-validation was used to prevent information leakage during model selection and to obtain a less biased estimate of generalization performance (inner loop: hyperparameter tuning/model selection; outer loop: performance evaluation) [47]. The outer loop fold number was determined by the smallest group size in each comparison to ensure sufficient training samples per outer fold: 10-fold cross-validation for BP vs HC (n=60 vs 99; smallest group n=60), 5-fold cross-validation for mood state vs HC analyses (n=20‐32 vs 97; smallest group n=20‐32), and 5-fold cross-validation for direct interstate comparisons (n=15‐27 vs 15‐27 after exclusion of overlapping participants). In each outer loop iteration, 1/k of the data was held out as the test fold (ie, 10% for k=10% and 20% for k=5). Within each outer training fold, undersampling with age and sex matching was performed 10 times [46,48], followed by inner loop cross-validation for hyperparameter optimization (Multimedia Appendix 1, section 6). This yielded 100 models for BP vs HC (10 folds × 10 subsamplings), 50 models for mood state vs HC analyses (5 folds × 10 subsamplings), and 50 models for interstate comparisons (5 folds × 10 subsamplings). Within the matched training subsamples, residual mean age differences were below 0.5 years for all comparisons with HC but were only partially reduced for the interstate comparisons involving MS/MX, reflecting their smaller participant pools (see Multimedia Appendix 1, section 9). For each participant in each outer test fold, predicted probabilities from the 10 age- and sex-matched undersampling iterations within that fold were averaged to yield a single diagnostic probability. Classification metrics were computed from the participant-level averaged out-of-fold probabilities: for the comparisons with HC, at the cut-point maximizing the Youden index, and for the comparisons between mood states, at a threshold of 0.5. Kernel density estimation was used to visualize distributions, and performance metrics (AUC, accuracy, sensitivity, specificity) were computed.

Feature importance was based on selection frequency across cross-validated runs and validated via permutation testing. This approach, focusing on selection frequency rather than average coefficients or weights, was adopted to identify generalizable acoustic and linguistic features across different cross-validation folds and subsamplings, following a previously established methodology [46]. The consistent selection of features across independent folds provides a more robust indicator of feature importance under this resampling design. Features with selection frequencies exceeding the 95th percentile of the permutation-based null distribution were deemed statistically significant (*P*<.05; see Multimedia Appendix 1, section 7 for details). For display in the *Results*, among the acoustic features meeting this significance criterion in each classification, we selected the single feature most strongly correlated with the diagnostic probability or with symptom severity. This permutation-based thresholding was used to account for multiple testing across the high-dimensional feature set within each classification task. For interstate comparisons, participants appearing in both groups were excluded to ensure sample independence. Because sample sizes for these comparisons were smaller than those for mood state vs HC analyses and statistical power was correspondingly lower, permutation testing was performed with additional model-level evaluation. In addition to permutation testing for feature selection significance, model-level permutation testing (100 iterations with label shuffling at the participant level) was performed to assess whether the observed AUC significantly exceeded chance. This dual approach provided statistical validation at both the feature and model levels.

### Statistical Analysis

Each analysis included all participants who had the data required by that analysis. Because every comparison used age- and sex-matched undersampling, participants with missing age or sex data could not be included in any analysis; this excluded 1 patient with BP (retaining 60 of 61 patients for BP vs HC and 20 of 21 patients in the MS/MX group). The mood state analyses additionally required at least 1 recording session with the complete 3-task battery so that each state-level feature vector reflected the same task composition across participants (32 patients with DS, 20 patients with MS/MX, and 26 patients with ES; 97 HC). Because the BP vs HC classification instead averaged each participant’s features across all recordings, the 2 HC participants without such a session contributed to that comparison (99 HC) but not to the mood state comparisons. No participant was excluded for any other reason.

Demographic comparisons between BP and HC used the Student *t* test or Mann-Whitney *U* test, depending on normality (Shapiro-Wilk test, *P*<.05). Categorical variables were analyzed using chi-square tests. For state-specific comparisons, nonparametric tests were selected a priori given the clinical nature of the outcome variables. Kruskal-Wallis tests were used for continuous variables, and chi-square tests were used for categorical variables, followed by post hoc pairwise comparisons using Dunn test with Bonferroni correction (*α*=.0083 for 6 pairwise comparisons among 4 groups). All statistical tests were 2-tailed (significance: *P*<.05).

Model stability was assessed via AUC distributions (median, mean, and SD) across cross-validation models. Significant features were further correlated with diagnostic probabilities and clinical scores using Pearson or Spearman correlation, based on data normality. False discovery rate correction (Benjamini-Hochberg, q<0.05) was applied within each analysis batch rather than across all tests jointly: correlations involving the acoustic features identified as significant in the classification analyses were corrected together (correlations with the diagnostic probability and with HAMD-17 and YMRS scores; m=203), and correlations involving the linguistic features were corrected within the corresponding linguistic analysis. To assess the stability of feature selection and the associated risk of overfitting, we quantified, across all resampling iterations, the distribution of the number of selected features, the per-feature selection frequency, the consistency of the coefficient sign, and the pairwise overlap (Jaccard index) of the selected feature sets, together with a chance-corrected stability index (Nogueira index) [49].

To examine potential confounding by medication, we performed a post hoc sensitivity analysis within patients (HC excluded) because all patients with BP were medicated and no group of unmedicated patients with BP was available. Medication was coded from clinical records as the presence or absence of each class (antipsychotics, mood stabilizers, antidepressants, and anxiolytics), with lithium examined separately. For each classification model, we tested whether the diagnostic probability and the primary acoustic marker differed by medication class and whether the marker was associated with total medication burden before and after adjustment for symptom severity (HAMD-17). The dose-response relationship was assessed in an exploratory manner using chlorpromazine and diazepam equivalents [50]. Full methodological detail and results are provided in Multimedia Appendix 1 (section 11).

### Ethical Considerations

This study was approved by the Institutional Review Board of Keio University School of Medicine (approval 20170388; protocol 2017-0388-23), and the study was conducted in accordance with the Declaration of Helsinki. All participants provided written informed consent after receiving a full explanation of the study and were recruited between 2017 and 2024. All data were deidentified, and audio recordings were stored on encrypted servers. Participants could withdraw their consent at any time. This study is reported in accordance with the Strengthening the Reporting of Observational Studies in Epidemiology (STROBE) guidelines. A completed STROBE checklist is provided as Checklist 1.

## Results

### Participant Characteristics

Our study included 61 patients with BP and 99 HC participants. The 2 groups were comparable in age and sex distribution (Table 1). Patients with BP exhibited significantly higher HAMD-17 and YMRS scores compared to HC (*P*<.001 for both). Repeated assessments (median 2 [IQR 2-3] visits overall, range 1‐5; median 3 [IQR 1-5] visits for patients with BP) identified 78 distinct mood state classifications across the 61 patients with BP (mood state group membership was defined on the sessions that met the analysis criteria, see Multimedia Appendix 1, section 15; some patients met criteria for different mood states at different visits): DS (n=32), MS/MX (n=20), and ES (n=26). For state-specific analyses, 1 qualifying visit was randomly selected per participant for each applicable mood state. Most patients in depressive and manic states exhibited mild to moderate symptom severity (Table 2). All enrolled patients with BP had already received pharmacological treatment. The majority received mood stabilizers (77%, 47/61) and antipsychotics (72.1%, 44/61). Approximately one-third (32.8%, 20/61) received antidepressants, and half (49.2%, 30/61) received anxiolytics. The most common treatment combination was mood stabilizers plus antipsychotics (34.4%, 21/61), followed by mood stabilizers alone (21.3%, 13/61). Concomitant anxiolytic use was observed in 49.2% (30/61) of patients overall. Detailed medication profiles are presented in Table 3.

**Table 1.** Overall comparison of sample characteristics of the 2 groups: patients with bipolar disorder (BP) and healthy control (HC) participants.^a^

Characteristic	BP	HC	*P* value
Number of participants, n	61	99	—^b^
Age^c^ (years), mean (SD)	48.3 (15.4)	51.7 (18)	.21
Sex (female), %	54.1	55.6	.98
HAMD-17^d^ total score, mean (SD)	11.1 (7.4)	1.2 (1.6)	<.001
YMRS^e^ total score, mean (SD)	5.5 (7)	0.2 (0.6)	<.001

a Group differences were assessed using 2-tailed tests (described in *Methods*).

b Not applicable.

c Age was available for 60 of the 61 patients with BP.

d HAMD-17: 17-item Hamilton Depression Rating Scale.

e YMRS: Young Mania Rating Scale.

**Table 2.** Comparison of state-specific characteristics of patients with bipolar disorder and healthy control (HC) participants^a^.

Characteristic	DS^b^	MS/MX^c^	ES^d^	HC	Overall *P* value
Number of participants, n	32	20	26	99	—^e^
Age^f^ (years), mean (SD)	45.1 (15.4)	54.2 (12.3)	49.2 (16.8)	51.7 (18)	.15
Sex (female), %	59.4	55	34.6	55.6	.23
HAMD-17^g^ total score, mean (SD)	14.9 (5.9)	11.2 (7.6)	4 (2.1)	1.2 (1.6)	<.001
YMRS^h^ total score, mean (SD)	2.6 (2.2)	14.3 (5.7)	1.3 (1.7)	0.2 (0.6)	<.001

a Group differences were assessed using 2-tailed tests (described in *Methods*).

b DS: depressive state.

c MS/MX: state with manic/mixed symptoms.

d ES: euthymic state.

e Not applicable.

f Age was available for 60 of the 61 patients with bipolar disorder.

g HAMD-17: 17-item Hamilton Depression Rating Scale.

h YMRS: Young Mania Rating Scale.

**Table 3.** Medication use patterns in patients with bipolar disorder (n=61).

Use pattern	Number of patients, n (%)
Medication class^a^	
Mood stabilizers	47 (77)
Antipsychotics	44 (72.1)
Anxiolytics	30 (49.2)
Antidepressants	20 (32.8)
Others^b^	52 (85.2)
Combination pattern^c^ and concomitant use of anxiolytics	
Mood stabilizers + antipsychotics	21 (34.4)
Concomitant anxiolytics^d^ (n/n)	10/21
Mood stabilizers alone	13 (21.3)
Concomitant anxiolytics (n/n)	6/13
Mood stabilizers + antipsychotics + antidepressants	9 (14.8)
Concomitant anxiolytics (n/n)	7/9
Antipsychotics alone	7 (11.5)
Concomitant anxiolytics (n/n)	1/7
Antipsychotics + antidepressants	7 (11.5)
Concomitant anxiolytics (n/n)	5/7
Mood stabilizers + antidepressants	4 (6.6)
Concomitant anxiolytics (n/n)	1/4

a A participant counted toward a medication class if that class was prescribed at any visit during the observation period; because classes overlap, percentages in this part of the table do not sum to 100%.

b The category “Others” mainly comprised medications for physical comorbidities.

c A list of all observed psychotropic combination patterns; the 6 patterns are mutually exclusive and account for all 61 patients.

d The proportion of patients within each pattern also receiving anxiolytics.

The results of Bonferroni-corrected comparisons vs HC were as follows: for HAMD-17, DS and MS/MX *P*<.001 and ES *P*=.002; for YMRS, DS and MS/MX *P*<.001 and ES *P*=.004. Between-state comparisons showed that HAMD-17 differed in all 3 pairs (DS vs ES, *P*<.001; DS vs MS/MX, *P*=.003; MS/MX vs ES, *P*=.003), with DS>MS/MX>ES. YMRS was highest in MS/MX (DS vs MS/MX and MS/MX vs ES, both *P*<.001), whereas it did not differ between DS and ES (*P*>.99). Age and sex did not differ between states.

### Machine Learning Classification Performance

The machine learning model distinguished BP from HC with high discrimination (AUC=0.916, 95% CI 0.854‐0.958; sensitivity 0.933, specificity 0.859; n=60 vs 99). The probability distribution curves (Figure 1A) showed clear group separation, indicating strong discriminative power. State-specific analyses yielded varying performance: DS vs HC (n=32 vs 97; AUC=0.805, 95% CI 0.730‐0.905), MS/MX vs HC (n=20 vs 97; AUC=0.848, 95% CI 0.814‐0.950), and ES vs HC (n=26 vs 97; AUC=0.874, 95% CI 0.833‐0.949), with ES achieving the highest classification performance among the point estimate AUCs. The corresponding probability distributions of each mood state compared with HC are shown in Figure 1B. Performance was consistent in direction across cross-validation iterations, although variability across folds and subsamplings was present (Figure 1C). By median AUC across resampled models, the BP vs HC classification ranked highest (median AUC=0.933 across 100 models [10 folds × 10 subsamplings]), followed by MS/MX (median AUC=0.908, across 50 models [5 folds × 10 subsamplings]), ES (median AUC=0.885, across 50 models [5 folds × 10 subsamplings]), and DS (median AUC=0.846, across 50 models [5 folds × 10 subsamplings]). IQRs were relatively narrow, indicating that performance was reproducible across resampled models within this cohort; however, because all estimates were derived from the same participants, this consistency does not by itself exclude overfitting. These classification results are summarized in Table 4.

To quantify the independent and incremental contribution of each modality, we repeated the BP vs HC classification using linguistic features only (n=211), acoustic features only (3473 candidate features; 6373 extracted acoustic features minus the 2900 volume-dependent features excluded before model training), and both combined. Each modality discriminated above chance (model-level permutation *P*≤.02). The acoustic-only classification (AUC 0.910) exceeded the linguistic-only classification (AUC 0.651), and the combined model (AUC 0.911) matched the acoustic-only model, indicating negligible incremental contribution from the linguistic features (Multimedia Appendix 1, section 12). Because the 2 modality-specific models drew on candidate feature sets of very different sizes, the difference between them is not a comparison at matched dimensionality (see *Limitations*). These modality-specific models were rerun with a fixed random seed; thus, the combined-model AUC of 0.911 differs from the primary estimate of 0.916 reported above by less than the seed-to-seed variability (SD ≤0.01). The same pattern was observed for the mood state comparisons, in which combining modalities changed the AUC by at most 0.012.

Direct interstate classification analyses, conducted with overlapping participants excluded to ensure sample independence, yielded mixed results. The DS vs ES comparison achieved an AUC of 0.760, which significantly exceeded chance level (permutation test, *P*=.03). In contrast, the MS/MX vs ES comparison yielded an AUC of 0.601 (*P*=.20), and the DS vs MS/MX comparison resulted in an AUC of 0.569 (*P*=.30), neither reaching statistical significance. No individual features exceeded the permutation-derived significance threshold at the feature level in any interstate comparison.

**Figure 1.**
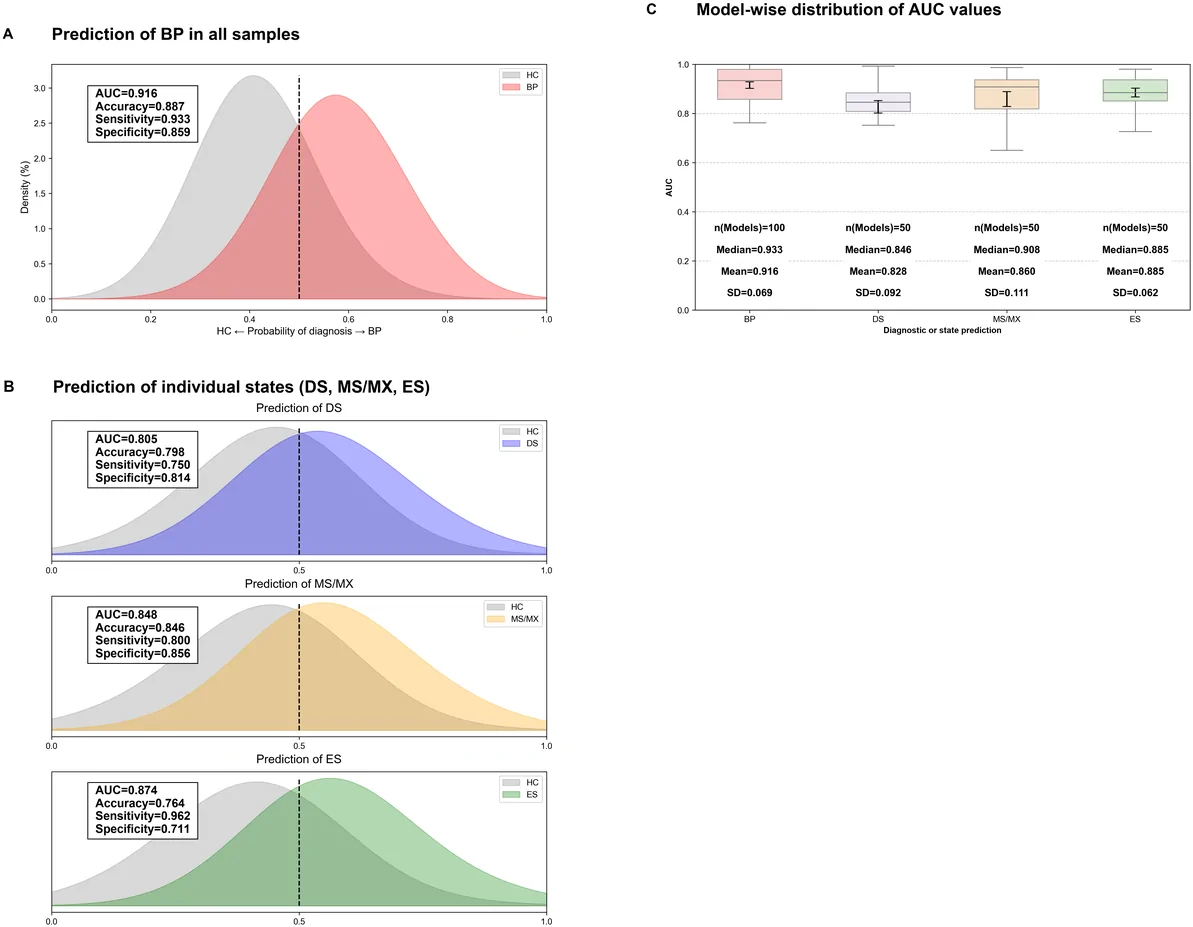
Diagnostic performance assessment of machine learning classification models. (A) Distribution of diagnostic probabilities for patients with bipolar disorder (BP) vs healthy controls (HC); the dashed line marks a probability of 0.5 for reference. The insets in (A) and (B) present the area under the curve (AUC) and the accuracy, sensitivity, and specificity at the cut-point that maximizes the Youden index. (B) Probability distributions for HC and BP mood states—depressive state (DS), state with manic/mixed symptoms (MS/MX), and euthymic state (ES). (C) AUC distributions by mood state: median (horizontal line), IQR (box margins), and 95% CI (error bars) from 1000 bootstrap samples; the median, mean, and SD of the AUC across these models are also indicated. n(Models) is the number of resampled models and not the participant sample size (as described in *Methods*).

**Table 4.** Classification performance for the comparisons with healthy controls and between mood states.^a^

Comparison	Number of participants, n^b^	AUC^c^ (95% CI)	Sensitivity	Specificity	Accuracy
BP^d^ vs HC^e^	60 vs 99	0.916 (0.854‐0.958)	0.933 (56/60)	0.859 (85/99)	0.887 (141/159)
DS^f^ vs HC	32 vs 97	0.805 (0.730‐0.905)	0.750 (24/32)	0.814 (79/97)	0.798 (103/129)
MS/MX^g^ vs HC	20 vs 97	0.848 (0.814‐0.950)	0.800 (16/20)	0.856 (83/97)	0.846 (99/117)
ES^h^ vs HC	26 vs 97	0.874 (0.833‐0.949)	0.962 (25/26)	0.711 (69/97)	0.764 (94/123)
DS vs ES	23 vs 17	0.760 (0.685‐0.936)	0.696 (16/23)	0.706 (12/17)	0.700 (28/40)
MS/MX vs ES	15 vs 21	0.601 (0.457‐0.819)	0.667 (10/15)	0.619 (13/21)	0.639 (23/36)
MS/MX vs DS	15 vs 27	0.569 (0.412‐0.759)	0.467 (7/15)	0.593 (16/27)	0.548 (23/42)

a Sensitivity, specificity, and accuracy were computed at the participant level, with the underlying participant counts in parentheses; thresholds and participant exclusions are described in the *Methods*, and metrics at a fixed threshold of 0.5 are given in Multimedia Appendix 1 (section 14).

b In each comparison, n is given for the first group followed by the second, and the first group is the positive class. Participants meeting criteria for more than 1 mood state were excluded from both groups in the comparisons between mood states; permutation *P* values are given in the *Results*.

c AUC: area under the receiver operating characteristic curve (cross-validated; as described in *Methods*).

d BP: bipolar disorder.

e HC: healthy control.

f DS: depressive state.

g MS/MX: state with manic/mixed symptoms.

h ES: euthymic state.

### Important Features and Clinical Correlations

The analyses identified significant features in tasks distinguishing BP from HC. Although both acoustic and linguistic features were entered as candidate predictors, the significant discriminative features were predominantly acoustic, indicating that the classification was driven primarily by acoustic features rather than by the combination of modalities per se. These features showed significant correlations with both diagnostic probabilities (Figures 2 and 3A-D) and clinical severity scores (Figure 3E-F). In the overall BP vs HC comparison, several acoustic features were strongly correlated with diagnostic probabilities (|ρ|=0.35‐0.85; Figure 2A). Feature selection was stable in direction and identified a reproducible core of features, although the exact selected set varied across resamples. For the combined BP vs HC model, a mean of 168 (SD 119) features was selected per resample, 116 features were selected in at least half of the resamples, the median per-feature coefficient-sign consistency was 1, and the mean pairwise Jaccard overlap of the selected sets was 0.48 (chance-corrected Nogueira index 0.56); across all models this overlap ranged from 0.18 (mood state comparisons) to 0.49. Because correlated acoustic features can substitute for one another across resamples, feature-level inference in this study is based on selection frequency rather than on any single model’s feature list (Multimedia Appendix 1, section 10).

**Figure 2.**
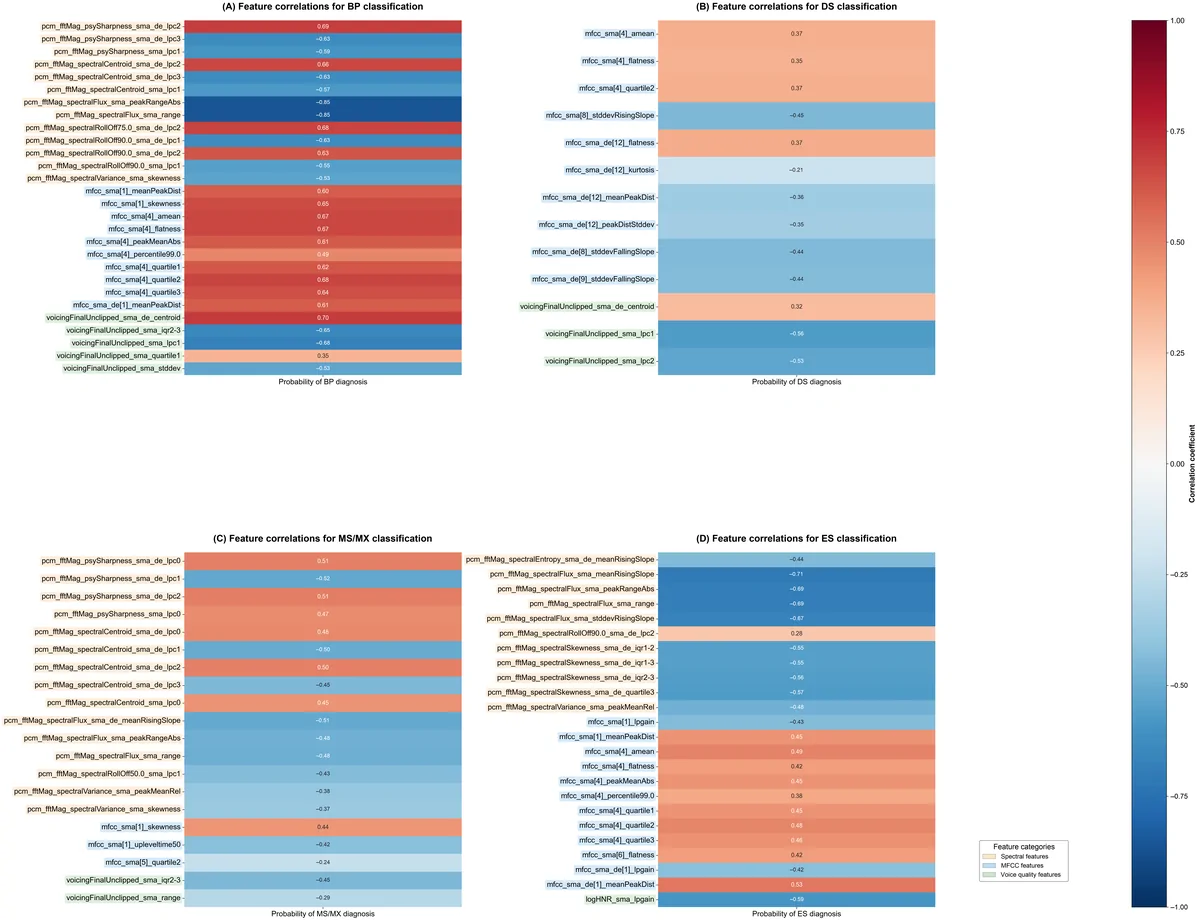
Correlation between acoustic features and diagnostic probability. Heat maps of Spearman correlations between acoustic features and diagnostic probability for bipolar disorder (BP) and its mood states (depressive state [DS], state with manic/mixed symptoms [MS/MX], and euthymic state [ES]). Features are grouped as spectral, mel-frequency cepstral coefficients (MFCCs), and voice quality. Color indicates the correlation coefficient (blue −1 to red 1). Each panel shows all features that were significant in the permutation-based selection and correlated with diagnostic probability after false discovery rate correction (q<0.05). (A) BP: 28 features, |ρ|=0.35‐0.85; (B) DS: 13 features, |ρ|=0.21‐0.56; (C) MS/MX: 20 features, |ρ|=0.24‐0.52; (D) ES: 24 features, |ρ|=0.28‐0.71.

**Figure 3.**
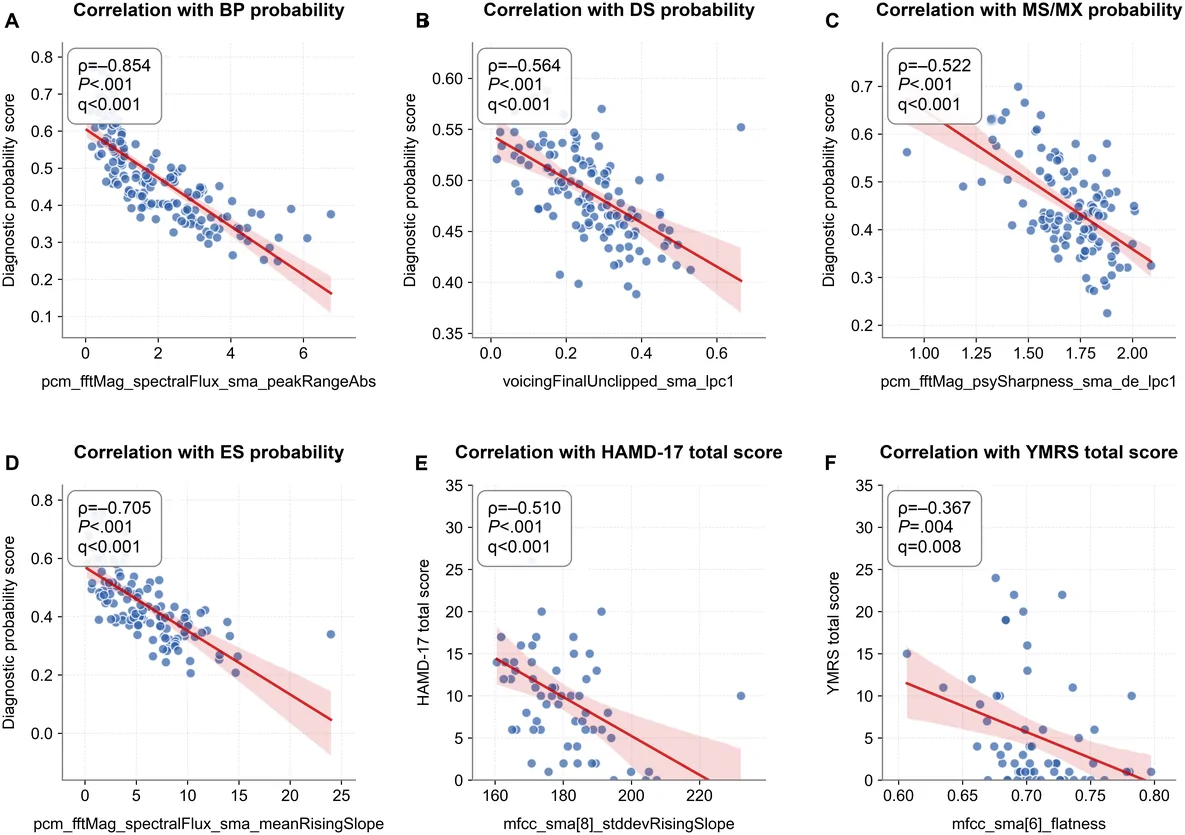
Acoustic feature correlations with diagnostic probabilities and symptom severity. Scatter plots with linear fits (red lines) and 95% CIs (pink) are included. For each comparison (bipolar disorder [BP], depressive state [DS], and state with manic/mixed symptoms [MS/MX], and euthymic state [ES] vs healthy controls [HC), 1 feature is shown: the feature most strongly correlated with the diagnostic probability (A-D) or with symptom severity (E-F; total score on the Hamilton Depression Rating Scale-17 [HAMD-17] and Young Mania Rating Scale [YMRS], respectively), selected from the significant acoustic features. (A) BP probability, pcm fftMag spectralFlux sma peakRangeAbs (ρ=−0.854, *P*<.001); (B) DS probability, voicingFinalUnclipped sma lpc1 (ρ=−0.564, *P*<.001); (C) MS/MX probability, pcm fftMag psySharpness sma delpc1 (ρ=−0.522, *P*<.001); (D) ES probability, pcm fftMag spectralFlux sma meanRisingSlope (ρ=−0.705, *P*<.001); (E) HAMD-17, mfcc sma 8 stddevRisingSlope (ρ=−0.510, *P*<.001); (F) YMRS, mfcc sma 6 flatness (ρ=−0.367, *P*=.004).

We categorized the features based on their patterns across HC comparison scenarios. Consistent with our operational definition, features identified as statistically significant in the ES vs HC comparison were classified as trait markers, as they were detectable even during remission periods with minimal symptoms. Features identified in the DS vs HC and MS/MX vs HC comparisons showed associations with each mood state when compared to HC. When overlapping participants were excluded to ensure sample independence, interstate classification showed that the DS vs ES comparison significantly exceeded chance (AUC=0.760, permutation *P*=.03), while the comparisons MS/MX vs ES (AUC=0.601, *P*=.20) and DS vs MS/MX (AUC=0.569, *P*=.30) did not reach significance. These results suggest that the identified features primarily reflect persistent disease characteristics, although the significant DS vs ES classification indicates that DS is partially distinguishable from ES at the model level despite no individual features reaching significance at the feature level. Nonetheless, these features were insufficient to distinguish between mood states involving MS/MX. This study distinguishes 2 categories of feature-level findings. First, statistically significant classification predictors: across the 4 comparisons with HC, acoustic features comprised 96% to 98% of the features identified as significant by the permutation-based selection with family-wise error correction (174 of 178 for BP, 118 of 121 for DS, 104 of 108 for MS/MX, and 123 of 125 for ES), and a small number of linguistic features (2-4 per comparison) also reached significance, including the ratio of the case particle “to,” which recurred across 3 comparisons, and the variability of sentiment expression (the full list is given in Multimedia Appendix 1, Table S13). However, these linguistic features were selected less stably than the acoustic features (Multimedia Appendix 1, section 10) and should be interpreted with caution. The modality composition of the significant features for each comparison is given in Table S12 in Multimedia Appendix 1.

Features associated with DS in comparison to HC primarily included higher-order MFCC features (8, 9, and 12; coefficients that describe finer variation of the spectral envelope across frequency), with temporal flatness metrics positively correlated with the diagnostic probability. Features associated with MS/MX in comparison to HC consisted mainly of spectral features related to variability. Trait markers included relatively lower-order MFCC features (1, 4, and 6; coefficients that describe the coarser overall shape of the spectral envelope) and specific spectral features consistently associated with ES diagnostic probability (Figure 2B-D).

Second, features correlated with symptom severity, a category distinct from the classification predictors above. HAMD-17 scores were inversely correlated with MFCC 8 variability (ρ=−0.51, *P*<.001, q<0.001), and YMRS scores were inversely correlated with MFCC 6 flatness (ρ=−0.37, *P*=.004, q=0.008) (Figure 3E-F). For the linguistic modality, anger-related parameters were significantly correlated with mania severity (anger maximum: ρ=0.32, *P*=.003, q=0.048; anger variability: ρ=0.36, *P*=.001, q=0.042). These anger-related linguistic parameters were not among the significant classification predictors but were correlated with severity, illustrating the distinction between the 2 categories. Modified speech examples using a WORLD vocoder-based approach and their corresponding acoustic feature values are presented in Multimedia Appendix 1 (section 8). Representative audio samples are provided in Multimedia Appendix 2 (Audio S1.1–S1.6).

Although the DS vs ES comparison achieved significant model-level performance (AUC=0.760, *P*=.03), no individual features exceeded the permutation-derived significance threshold at the feature level in the interstate comparisons.

In the medication sensitivity analysis, for the BP vs HC, DS vs HC, and MS/MX vs HC models, the diagnostic probability was not associated with any medication class, the primary marker did not differ by any medication class (no item significant after false discovery rate correction), and the marker was not correlated with total medication burden either before or after adjustment for symptom severity. Mood stabilizers and lithium likewise showed no significant association with the marker or the diagnostic probability. In contrast, for the ES vs HC model, although the primary marker itself did not differ by medication class, the diagnostic probability was higher in antipsychotic users (Cohen *d*=1.17, *P*=.005), and the marker remained correlated with total medication burden after adjustment for symptom severity (partial ρ=–0.45, *P*=.02). Thus, discrimination of ES could not be fully separated from medication.

## Discussion

### Principal Findings

This study investigated acoustic and linguistic features as potential markers for BP by integrating linguistic and acoustic features through NLP and speech analysis. This study makes four main contributions: (1) the model achieved high diagnostic discrimination for BP vs HC (AUC=0.916) and for each mood state vs HC (AUC=0.805‐0.874); (2) we identified disease-associated features that function as trait markers, remaining detectable even during remission in the ES vs HC comparison; (3) we identified features associated with each mood state (DS, MS/MX) when compared with HC; and (4) regarding interstate comparisons, the DS vs ES comparison significantly exceeded chance (AUC=0.760, *P*=.03), while other interstate comparisons did not reach significance.

First, regarding diagnostic performance, our model achieved higher discrimination (AUC=0.916) than the pooled classification accuracy (~0.77) reported in prior meta-analyses [21], likely reflecting the strong discriminative contribution of acoustic features, which were the predominant significant predictors in our models. Despite being the largest subgroup, the DS cohort had the lowest discrimination, possibly reflecting greater heterogeneity. In contrast, the ES classification achieved the highest state-specific point-estimate AUC, suggesting that trait markers in BP are more stable and detectable than previously assumed.

Notably, regarding feature modality, across the 4 HC comparison scenarios (BP vs HC, DS vs HC, MS/MX vs HC, and ES vs HC), the proportion of significant features that were acoustic ranged from 96% to 98% (BP vs HC, 97.8%; DS vs HC, 97.5%; MS/MX vs HC, 96.3%; ES vs HC, 98.4%), consistently exceeding that of significant linguistic features. These results suggest that speech delivery patterns may be more diagnostically informative than speech content alone. Modality-specific analyses confirmed that acoustic features alone achieved diagnostic performance essentially identical to that of the combined model (BP vs HC: AUC 0.910 vs 0.911), whereas linguistic features alone performed substantially worse (AUC 0.651), indicating that classification was driven by acoustic features, with little incremental contribution from linguistic features (Multimedia Appendix 1, section 12). A small number of linguistic features also reached significance across these comparisons (Multimedia Appendix 1, Tables S12 and S13), although they were selected less stably; these concern the classification predictors and are distinct from the features correlated with symptom severity.

These findings raise the question of why acoustic features show greater diagnostic utility. While the neurobiological mechanisms linking mood dysregulation to alterations in speech production remain incompletely understood, existing research suggests that dysfunction in basal ganglia–thalamocortical circuits involved in mood regulation may be associated with psychomotor symptoms, particularly speech delays and prosodic changes [51].

Second, regarding trait markers detectable during remission, features classified as trait markers for BP, including those present during remission periods, comprised low-order MFCC features, harmonics-to-noise ratio (HNR), and spectral features, indicating persistent alterations in speech. Among MFCC features, MFCC 4–related parameters were predominant, with MFCC 1 and MFCC 6 being more prominent than in the DS group. This pattern aligns with prior findings of positive correlations between mean MFCC 4 values and unipolar depression severity [52]. An inverse correlation was observed between logHNR and diagnostic probability, with BP groups showing significantly lower values compared to HC. Reduced HNR values suggest less regular vocal fold vibration and increased noise contamination, resulting in less distinct vocalizations, potentially reflecting characteristic acoustic features of BP [53]. Parameters related to spectral skewness also emerged as frequent markers, underscoring the diagnostic utility of spectral distribution asymmetry in BP. These results are consistent with a previously reported model incorporating MFCC 4, spectral skewness, and logHNR, which achieved an 80% AUC in identifying geriatric depression, suggesting that shared acoustic features may reflect common psychomotor effects rather than disorder-specific signatures [54]. Because individual coefficients other than MFCC 0 and MFCC 1 do not carry an established physical meaning, these comparisons with prior work are made at the level of coefficient order (low vs high) rather than as an exact match of individual coefficient indices.

Notably, several features identified in the ES vs HC comparison overlapped with those identified in DS vs HC and MS/MX vs HC comparisons, consistent with their interpretation as persistent disease-associated markers. However, each mood state comparison also identified distinct features, suggesting that while a core set of acoustic markers characterizes BP across states, additional features may be associated with specific mood states when compared to healthy individuals.

Third, regarding state-associated features, features associated with DS in comparison to HC primarily comprised higher-order MFCC features, particularly MFCC 12, MFCC 8, and MFCC 9. In the present study, the flatness of the MFCC 12 time series (mfcc sma de 12 flatness) was positively correlated with diagnostic probability. Our study uniquely identified an inverse correlation between MFCC 12 variability and DS diagnostic probability. Because MFCC 12 is a higher-order coefficient describing fine detail of the spectral envelope, its temporal flatness (little change over the course of the utterance) suggests that this fine spectral detail remains relatively static over time, a pattern that may reflect the reduced articulatory and spectral variation associated with psychomotor retardation in bipolar depression.

Features associated with MS/MX in comparison to HC were predominantly characterized by spectral features. Prior research supports the association of YMRS scores with spectral flux (related to conversational clarity) and psychoacoustic sharpness [20]. However, correlations between spectral features and the MS/MX diagnostic probabilities were inconsistent in direction, possibly reflecting the complex vocal modulations seen in manic states. For example, increased spectral flux variability, which reflects rapid changes in spectral energy distribution, may correspond to the pressure of speech and variable vocal intensity characteristic of manic episodes, while the inconsistent direction of these correlations may reflect the heterogeneity of manic presentations, ranging from euphoric to irritable subtypes. Interstate comparisons involving MS/MX are considered below. Further research with larger samples and more refined mood state definitions is warranted to clarify these associations.

Fourth, regarding interstate classification, when participant overlap between groups was excluded to ensure sample independence, the DS vs ES classification achieved significant above-chance performance (AUC=0.760, permutation *P*=.03). This result suggests that features associated with DS may capture not only persistent disease characteristics but also acoustic and linguistic patterns partially specific to DS. Although no individual features exceeded the permutation-derived threshold at the feature level, the significant model-level performance indicates that the combination of subtle changes across multiple features collectively enables the partial discrimination of DS from ES. In contrast, interstate classification involving MS/MX (MS/MX vs ES: AUC=0.601, *P*=.20) did not reach statistical significance, suggesting that features associated with MS/MX may primarily reflect disease-related characteristics. The inability to distinguish MS/MX from other mood states may reflect the clinical heterogeneity of manic presentations, which range from euphoric to irritable subtypes, and the relatively small sample size (n=15 after the exclusion of overlapping participants). Furthermore, DS vs MS/MX classification (AUC=0.569, *P*=.30) also failed to reach significance, indicating that while DS was partially distinguishable from ES, the features identified in this study were insufficient to discriminate between DS and MS/MX. This may reflect the fact that both states are distinguished from HC through partially overlapping acoustic features, making direct differentiation between them more challenging. Further validation in larger samples is warranted.

We examined potential medication confounding from several angles. The diagnostic probabilities and the primary acoustic markers were not associated with medication class or with total medication burden, and no association survived adjustment for symptom severity, with the exception of ES. Dose-response analyses using chlorpromazine and diazepam equivalents were exploratory and are not interpretable (see *Limitations*). For DS, MS/MX, and BP overall, we therefore found no evidence that the voice findings are attributable to pharmacological treatment rather than to the disorder itself, although a treatment contribution cannot be excluded. However, in ES, a residual association with antipsychotic use was observed, which we address below as a limitation.

### Limitations

Several limitations should be acknowledged. First, the study did not include comparisons with other psychiatric disorders, including major depressive disorder (MDD) and schizophrenia. Consequently, it remains unclear whether the identified features represent BP-specific markers or reflect broader psychopathological indicators. Future research examining BP vs MDD comparisons, particularly during depressive episodes, represents a critical next step toward clinical application. Second, the relatively small sample sizes, particularly after mood state stratification (DS: n=32, MS/MX: n=20, ES: n=26), in relation to the large feature set (3473 acoustic and 211 linguistic candidate features) may increase the risk of overfitting despite the use of LASSO regularization. LASSO nonetheless reduced the effective dimensionality of the fitted models substantially: in the BP vs HC model, a mean of 168 (SD 119) features was retained per resample, and 116 features were retained in at least half of the resamples. Even so, the ratio of candidate features to participants remained high, and the present analyses should therefore be regarded as exploratory and hypothesis-generating; the performance estimates reported here are best interpreted as provisional values requiring confirmation in larger samples. In addition, because the candidate acoustic and linguistic feature sets differed markedly in size (3473 vs 211), the performance gap between the acoustic-only and linguistic-only models cannot be fully separated from the larger search space available to the acoustic models; by contrast, the near-identical performance of the combined and acoustic-only models is a nested comparison and is unaffected by this asymmetry. The small subgroup sizes may have been particularly consequential for the interstate comparisons, where sample sizes were further reduced after excluding overlapping participants to ensure sample independence (DS vs ES: n=23 vs 17; MS/MX vs ES: n=15 vs 21; DS vs MS/MX: n=27 vs 15), limiting the statistical power to detect within-diagnosis differences. The nested cross-validation and repeated undersampling procedures were implemented to mitigate this concern, but larger sample sizes would strengthen the robustness of our findings. Moreover, participants excluded due to overlap may represent a clinically distinct subpopulation who experienced multiple mood states, which may limit the generalizability of the interstate findings. Third, although the single-language design minimized cross-linguistic variability, it may limit generalizability. Fourth, age- and sex-matched undersampling was applied to the training data only, and models were evaluated on all participants in each held-out test fold. Residual age differences within the matched training subsamples were negligible for the comparisons with HC but only partially reduced for the interstate comparisons involving MS/MX (Multimedia Appendix 1, section 9); because the test folds retained the age structure of the underlying cohorts (for example, patients in DS were on average 6.7 years younger than HC participants), age-related variance in the evaluation data cannot be fully excluded. In addition, the 4 comparisons with HC shared the same control participants, and 19 patients contributed to more than 1 mood state group; the comparisons are therefore not mutually independent, although observations are independent within each comparison. Fifth, as all patients were undergoing pharmacological treatment, the potential influence of medication on speech and language features cannot be fully excluded. Psychotropic medications are known to affect arousal, psychomotor activity, and cognitive processing, any of which may be reflected in vocal characteristics and language use. Because all patients with BP were medicated, whereas HC participants were not, some of the observed acoustic differences may reflect a combination of disease-related and treatment-related effects. Future studies including unmedicated patients or explicitly modeling medication class, dose, and duration will be needed to disentangle these factors. Sixth, operating-point metrics reported at the Youden index cut-point are selected on the pooled out-of-fold probabilities and may therefore be optimistic; metrics at a prespecified threshold of 0.5 are provided in Multimedia Appendix 1 (section 14). Seventh, all performance estimates were obtained by internal resampling within a single cohort, and no independent external validation was performed. Models validated only internally typically show optimism when applied to data collected under different recording, clinical, and demographic conditions. The AUCs reported here should therefore be read as internal estimates rather than as evidence of transportable diagnostic accuracy, and confirmation in an independent cohort is required before any claim of clinical utility. Finally, set-level feature selection stability was moderate for the BP vs HC model and low for the mood state comparisons (mean pairwise Jaccard 0.18-0.29), consistent with the smaller sample sizes of the latter; feature-level results should therefore be interpreted at the level of selection frequency and effect direction rather than as a fixed feature list.

Medication analyses have limitations. All patients were medicated, and no unmedicated bipolar group existed; therefore, diagnosis and medication could not be separated within a single model, and analyses were restricted to within-patient comparisons. Medication was not randomly assigned, adjustment was limited to measured severity, and subgroups were small (20-60 patients per model); thus, residual confounding cannot be excluded, and nonsignificant results indicate that an effect was undetected rather than absent. The association of ES with antipsychotic use warrants caution because overt symptoms are minimal in this state and discrimination from HC may have depended in part on being medicated. Dose-response analyses were exploratory because daily doses could be extracted for only a minority of prescriptions; class-based analyses are unaffected.

External validation in an independent cohort is the principal next step for this work. Recruitment for the present cohort has been completed, and efforts are underway to establish a separate prospective sample in which the models reported here can be evaluated without refitting.

### Conclusion

In conclusion, this study indicates the potential of automated speech and language analysis as an objective tool for BP diagnosis. The identification of persistent markers detectable across mood states, combined with high diagnostic discrimination (AUC=0.916), provides preliminary support for developing objective diagnostic tools. Acoustic features contributed substantially more than linguistic features to classification performance, supporting their diagnostic value. Notably, disease-associated speech patterns were detectable during euthymia. However, ES diagnostic probabilities were higher in antipsychotic users (Cohen *d*=1.17), so these patterns cannot presently be attributed to the disorder rather than to its treatment, and their utility for screening requires confirmation in unmedicated patients. Although DS showed partial distinguishability from ES at the model level (AUC=0.760, *P*=.03), the overall limited ability to distinguish between mood states within BP represents a significant limitation for clinical monitoring applications. Further validation in independent external cohorts and in larger, more diverse populations, as well as comparisons with other psychiatric conditions, remains essential to establish clinical utility.

## Supplementary material

10.2196/98094 Multimedia Appendix 1 Supplementary methods and results.

10.2196/98094 Multimedia Appendix 2 Representative audio samples of vocoder-based acoustic modifications. Audio S1.1: Japanese control sample (basic vocoder reconstruction); Audio S1.2: Japanese depression-like modification; Audio S1.3: Japanese mania-like modification; Audio S1.4: English control sample; Audio S1.5: English depression-like modification; Audio S1.6: English mania-like modification.

10.2196/98094 Checklist 1 STROBE checklist.

## Abbreviations

AUC: Area under the curve
BP: bipolar disorder
COGNISTAT: Neurobehavioral Cognitive Status Examination
DS: depressive state
DSM-5: Diagnostic and Statistical Manual of Mental Disorders, 5th Edition
ES: euthymic state
HAMD-17: 17-item Hamilton Depression Rating Scale
HC: healthy control
HNR: harmonics-to-noise ratio
ICD-11: International Classification of Diseases, 11th Revision
LASSO: least absolute shrinkage and selection operator
logHNR: logarithmic harmonic-to-noise ratio
MDD: major depressive disorder
MFCC: mel-frequency cepstral coefficients
MINI: Mini-International Neuropsychiatric Interview
MMSE: Mini-Mental State Examination
MS/MX: state with manic/mixed symptoms
NLP: natural language processing
STROBE: Strengthening the Reporting of Observational Studies in Epidemiology
UNDERPIN: Understanding Psychiatric Illness through Natural Language Processing
YMRS: Young Mania Rating Scale
