# Supplementary material for: Machine Learning Classification of Bipolar Disorder Across Mood States Using Natural Language Processing and Speech Analysis: Observational Study
Source: J Med Internet Res. 2026 Nov 1;28:e98094. doi: 10.2196/98094 (PMC13723951; doi:10.2196/98094)
Supplement: Supplementary file 1 [file jmir-v28-e98094-s003.pdf]

STROBE Statement—checklist of items that should be included in reports of observational studies

	Item No	Recommendation	Location in manuscript
**Title and abstract**	1	(*a*) Indicate the study's design with a commonly used term in the title or the abstract	Title; Abstract
		(*b*) Provide in the abstract an informative and balanced summary of what was done and what was found	Abstract
**Introduction**			
Background/ rationale	2	Explain the scientific background and rationale for the investigation being reported	Introduction
Objectives	3	State specific objectives, including any prespecified hypotheses	Introduction (final paragraph)
**Methods**			
Study design	4	Present key elements of study design early in the paper	Introduction (final paragraph); Methods – Participants; Methods – Machine Learning Classification
Setting	5	Describe the setting, locations, and relevant dates, including periods of recruitment, exposure, follow-up, and data collection	Methods – Participants; Methods – Ethical Considerations
Participants	6	(*a*) *Cohort study*—Give the eligibility criteria, and the sources and methods of selection of participants. Describe methods of follow-up<br>*Case-control study*—Give the eligibility criteria, and the sources and methods of case ascertainment and control selection. Give the rationale for the choice of cases and controls<br>*Cross-sectional study*—Give the eligibility criteria, and the sources and methods of selection of participants	Methods – Participants
		(*b*) *Cohort study*—For matched studies, give matching criteria and number of exposed and unexposed<br>*Case-control study*—For matched studies, give matching criteria and the number of controls per case	Methods – Machine Learning Classification (age- and sex-matched undersampling); Multimedia Appendix 1, Section 9
Variables	7	Clearly define all outcomes, exposures, predictors, potential confounders, and effect modifiers. Give diagnostic criteria, if applicable	Methods – Clinical Assessment and Data Collection; Methods – Data Collection and Feature Extraction Overview; Methods – Machine Learning Classification
Data sources/ measurement	8*	For each variable of interest, give sources of data and details of methods of assessment (measurement). Describe comparability of assessment methods if there is more than one group	Methods – Clinical Assessment and Data Collection; Methods – Data Collection and Feature Extraction Overview; Methods

			– Natural Language Processing Analyses; Methods – Speech Analyses
Bias	9	Describe any efforts to address potential sources of bias	Methods – Machine Learning Classification; Methods – Statistical Analysis; Discussion – Limitations
Study size	10	Explain how the study size was arrived at	Methods – Machine Learning Classification (a priori power analysis)
Quantitative variables	11	Explain how quantitative variables were handled in the analyses. If applicable, describe which groupings were chosen and why	Methods – Clinical Assessment and Data Collection; Methods – Machine Learning Classification
Statistical methods	12	(*a*) Describe all statistical methods, including those used to control for confounding	Methods – Machine Learning Classification; Methods – Statistical Analysis
		(*b*) Describe any methods used to examine subgroups and interactions	Methods – Machine Learning Classification (mood-state comparisons); Methods – Statistical Analysis
		(*c*) Explain how missing data were addressed	Methods – Statistical Analysis
		(*d*) *Cohort study*—If applicable, explain how loss to follow-up was addressed<br>*Case-control study*—If applicable, explain how matching of cases and controls was addressed<br>*Cross-sectional study*—If applicable, describe analytical methods taking account of sampling strategy	Methods – Machine Learning Classification (one aggregated data point per participant; age- and sex-matched undersampling)
		(*e*) Describe any sensitivity analyses	Methods – Statistical Analysis (medication); Methods – Machine Learning Classification (modality-specific analyses); Multimedia Appendix 1, Sections 11–14

Continued on next page

**Results**			
Participants	13*	(a) Report numbers of individuals at each stage of study—eg numbers potentially eligible, examined for eligibility, confirmed eligible, included in the study, completing follow-up, and analysed	Methods – Participants; Methods – Statistical Analysis; Results – Participant Characteristics; Table 1
		(b) Give reasons for non-participation at each stage	Methods – Statistical Analysis
		(c) Consider use of a flow diagram	Not applicable (participant numbers are reported in the text and Table 1)
Descriptive data	14*	(a) Give characteristics of study participants (eg demographic, clinical, social) and information on exposures and potential confounders	Results – Participant Characteristics; Tables 1–2
		(b) Indicate number of participants with missing data for each variable of interest	Methods – Statistical Analysis; Table 1 (note)
Outcome data	15*	*Cross-sectional study*—Report numbers of outcome events or summary measures	Results – Machine Learning Classification Performance; Table 4; Figure 1
Main results	16	(*a*) Give unadjusted estimates and, if applicable, confounder-adjusted estimates and their precision (eg, 95% confidence interval). Make clear which confounders were adjusted for and why they were included	Results – Machine Learning Classification Performance; Results – Important Features and Clinical Correlations (medication); Tables 3–4; Figure 1
		(*b*) Report category boundaries when continuous variables were categorized	Methods – Machine Learning Classification (HAMD-17 and YMRS cutoffs)
		(*c*) If relevant, consider translating estimates of relative risk into absolute risk for a meaningful time period	Not applicable
Other analyses	17	Report other analyses done—eg analyses of subgroups and interactions, and sensitivity analyses	Results – Machine Learning Classification Performance; Results – Important Features and Clinical Correlations; Figures 2–3; Multimedia Appendices 1–2
**Discussion**			
Key results	18	Summarise key results with reference to study objectives	Discussion
Limitations	19	Discuss limitations of the study, taking into account sources of potential bias or imprecision. Discuss both direction and magnitude of any potential bias	Discussion – Limitations
Interpretation	20	Give a cautious overall interpretation of results considering objectives, limitations, multiplicity of analyses, results from similar studies, and other relevant evidence	Discussion; Conclusion
Generalisability	21	Discuss the generalisability (external validity) of the study results	Discussion – Limitations
**Other information**			

Funding	22	Give the source of funding and the role of the funders for the present study and, if applicable, for the original study on which the present article is based	Funding

*Give information separately for cases and controls in case-control studies and, if applicable, for exposed and unexposed groups in cohort and cross-sectional studies.

Note: An Explanation and Elaboration article discusses each checklist item and gives methodological background and published examples of transparent reporting. The STROBE checklist is best used in conjunction with this article (freely available on the Web sites of PLoS Medicine at http://www.plosmedicine.org/, Annals of Internal Medicine at http://www.annals.org/, and Epidemiology at http://www.epidem.com/). Information on the STROBE Initiative is available at www.strobe-statement.org.
