# Supplementary material for: Machine Learning Classification of Bipolar Disorder Across Mood States Using Natural Language Processing and Speech Analysis: Observational Study
Source: J Med Internet Res. 2026 Nov 1;28:e98094. doi: 10.2196/98094 (PMC13723951; doi:10.2196/98094)
Supplement: Supplementary file 2 [file jmir-v28-e98094-s001.docx]

**Multimedia Appendix 1. Supplementary Methods and Results**

This supplementary document provides extended methodological details and additional results supporting the main manuscript titled 'Machine Learning Classification of Bipolar Disorder Across Mood States Using Natural Language Processing and Speech Analysis: Observational Study.' It comprises Supplementary Methods (Sections 1 to 7) and Supplementary Results (Sections 8 to 15), the latter including the medication sensitivity analysis (Section 11), the modality-specific classification analysis (Section 12), and the modality composition of the significant features (Section 13), which are referenced in the main text.

**Supplementary Methods**

*Section 1. Sentiment Analysis*

The Japanese BERT (Bidirectional Encoder Representations from Transformers) model (bert-base-japanese-v3)[1,2], pre-trained on the Japanese subset of the CC-100 (Common Crawl-100) dataset and Japanese Wikipedia[3], was fine-tuned using the WRIME dataset (a dataset of writers’ and readers’ emotion intensity ratings; approximately 35,000 Japanese social networking service texts) to assess emotional states and mood variability in bipolar disorder (BP)[4,5]. The texts were annotated with Plutchik’s eight emotions (joy, sadness, anticipation, surprise, anger, fear, disgust, and trust) and five-level sentiment polarity (strongly positive, positive, neutral, negative, strongly negative) for a comprehensive assessment of emotional valence [6]. For each participant’s sentence, we extracted the emotion intensity scores for all eight emotions and the sentiment polarity scores. We then computed descriptive statistics (mean, variance, standard deviation, maximum, minimum) across all sentences within each task to quantify the overall emotional profile.

*Section 2. Morphological Analysis*

Using GiNZA v5.2.0, a spaCy-based Japanese-language model with part-of-speech tagging capabilities [7], we analyzed grammatical and lexical distributions within the transcribed text. Measurements included the total morpheme count and morpheme-to-sentence ratio to assess speech volume and complexity. We examined linguistic features previously linked to depression, including the frequency of personal pronouns (first-, second-, and third-person), temporal references (including past-tense verbs and time expressions), and the distribution of various parts of speech (verbs, adverbs, demonstratives, nouns, and particles). These metrics were selected to capture both linguistic complexity and clinically relevant content features in the discourse.

*Section 3. Syntactic Analysis*

The syntactic analysis employed GiNZA’s dependency parsing capabilities to investigate structural relationships within clinical discourse. The analysis comprised three components:

a. Dependency tree metrics: We analyzed dependency distances (maximum, minimum, mean, variance, standard deviation) and breadth (number of dependent tokens per head), providing insights into speech branching patterns.

b. Dependency types: We examined conjunctive relationships, parallel clause structures, auxiliary and complement clauses, and subject-predicate relationships, including tracking the diversity and changes in subject types across utterances. We also analyzed the distribution and variation of root verbs, which carry significant semantic weight in clinical narratives.

c. Inter-sentential relationships: We analyzed sentence-initial elements (including conjunctions and pronouns), sentence types (declarative, interrogative, emphatic, and imperative), and cohesive expressions linking sentences to evaluate inter-sentential cohesion.

*Section 4.* *Acoustic Feature Analysis (openSMILE)*

We utilized openSMILE, an open-source toolkit for audio feature extraction, with the ComParE 2016 feature set [8,9]. Following the feature framework used in the main manuscript, the 6,373 extracted acoustic features were organized into three categories: (1) mel-frequency cepstral coefficients (MFCCs); (2) spectral features (for example, formants, spectral bands, and spectral flux); and (3) voice-quality features (jitter, shimmer, and harmonics-to-noise ratio). Volume-dependent prosodic features (for example, intensity and loudness) were excluded from the analysis because the microphone-to-mouth distance was not fixed and could not be standardized. Specifically, this exclusion removed four openSMILE feature families (pcm_RMSenergy, audspec_lengthL1norm, audspecRasta_lengthL1norm, and audSpec_Rfilt; 2,900 of the 6,373 extracted features), leaving 3,473 acoustic features as candidate predictors. In the classification pipeline, the exclusion was applied before model training, within each cross-validation fold, after a distribution-shape filter that removed features with extreme skewness, extreme kurtosis, or low variance in the training subsample. These features were complemented by statistical functionals (means, standard deviations, extremes, percentiles) to capture the temporal dynamics of speech. This feature set has been used in diverse paralinguistic contexts and was selected for its coverage of speech characteristics relevant to mood states.

*Section 5.* *Acoustic Feature Manipulation Using the WORLD Vocoder*

Note: These modifications are based on features associated with each mood state when compared to healthy controls (HC). When participant overlap between groups was excluded to ensure sample independence, inter-state classification showed that the depressive state (DS) vs. euthymic state (ES) comparison significantly exceeded chance (AUC = 0.760, permutation *p* = 0.030), while other inter-state comparisons did not reach significance (state with manic/mixed symptoms (MS/MX) vs. ES: AUC = 0.601, *p* = 0.198; DS *vs.* MS/MX: AUC = 0.569, p = 0.297). These features likely reflect a combination of persistent disease characteristics and state-associated patterns, with partial utility for distinguishing depressive from euthymic states.

To illustrate how the identified acoustic features might manifest in speech, we created modified speech samples using a WORLD vocoder-based approach[10]. Because manipulating individual acoustic features in isolation is technically challenging, we modified speech parameters based on our classification model findings.

We collected one natural conversational-like phrase (e.g., “Do you know any cafes around here?”) from each of 16 healthy volunteers (9 males, 7 females; age range: 23–48 years) who were not included in the UNDERPIN dataset, resulting in 16 unique speech samples. All participants were fluent Japanese speakers. The age distribution included 35.2% in their 20s, 53.0% in their 30s, and 11.8% in their 40s. Recordings were captured in WAV format at 16 kHz/16-bit resolution.

Three versions were created for each sample: (1) Reconstruction only (control), (2) Depression-like modification, and (3) Mania-like modification. The control samples were created using basic vocoder reconstruction to account for potential artifacts.

To validate our modifications, we extracted key acoustic features using openSMILE 3.0 before and after modification to confirm the appropriate changes in the targeted features. For depression-like modifications *vs*. control and mania-like modifications *vs*. control, we conducted Wilcoxon signed-rank tests to evaluate the significance of these acoustic feature changes.

To explore cross-linguistic generalizability, we included supplementary audio files that apply the same modification protocol to English speech samples from the CMU ARCTIC database[11].

*Section 6.* *Model Selection and Hyperparameter Optimization*

This section provides detailed descriptions of the LASSO model selection procedure and hyperparameter optimization strategy summarized in the main manuscript's Machine Learning Classification section.

LASSO was selected for its ability to simultaneously perform feature selection and regularization, addressing concerns about feature redundancy and high collinearity in our high-dimensional feature set (211 linguistic and 3,473 candidate acoustic features; of the 6,373 extracted acoustic features, 2,900 volume-dependent features were excluded before model training, as described in Section 4). LASSO regularization helps mitigate multicollinearity issues by automatically selecting a subset of features and setting coefficients of redundant features to zero[12]. We did not employ additional dimensionality reduction techniques (e.g., principal component analysis or correlation-based filtering) as LASSO’s built-in feature selection was deemed sufficient.

LASSO-regularized logistic regression was selected as the primary classifier for its dual capacity for feature selection and classification, which aligns with our study’s goal of identifying interpretable biomarkers rather than solely maximizing predictive accuracy. While comparison with non-linear classifiers (e.g., SVM, random forests, XGBoost) could provide additional insights into classification stability, the interpretability of LASSO coefficients and the direct identification of contributing features were prioritized for this exploratory biomarker study.

Prior to regularization, all features were z-score standardized to ensure comparability across modalities. To optimize the regularization parameter λ in the inner loop, we first calculated λmax (the minimum λ that reduces all coefficients to zero) [13]. We then generated 25 logarithmically spaced λ values ranging from 10^−6^ to λmax, based on previous studies[12]. The optimal λ was determined using the one-standard-error rule: among the 25 candidate values, we selected the largest λ within one standard error of the λ yielding the minimum prediction error (or equivalently, the highest AUC) during inner-loop cross-validation[13].

*Section 7. Feature Importance Assessment and Statistical Validation*

We assessed feature importance based on the selection frequency in the cross-validated LASSO logistic regression models using the scikit-learn LASSO implementation. Each feature could be selected a maximum of 100 times (10-fold cross-validation × 10 subsamplings) for BP *vs*. HC, 50 times (5-fold cross-validation × 10 subsamplings) for mood state *vs*. HC classifications, or 50 times (5-fold cross-validation × 10 subsamplings) for direct inter-state comparisons (DS *vs*. ES, MS/MX *vs*. ES, DS *vs*. MS/MX).

We applied permutation testing with 100 random shuffles of diagnostic labels to establish a null distribution. Shuffling was performed at the participant level, and in each permutation only the maximum selection count across all candidate features was retained, so that the null distribution consisted of 100 per-permutation maxima (a max-statistic permutation procedure). Features with selection frequencies exceeding the 95th percentile of this distribution (*p* < 0.05) were considered statistically significant. Because a single threshold derived from the distribution of per-permutation maxima was applied to every feature, this procedure controls the family-wise error rate across the full candidate feature set within each classification task[14].

**Supplementary Results**

*Section 8. Acoustic Feature Manipulation Results*

Representative examples of the acoustic feature modifications are provided as supplementary audio files:

- **Audio S1.1**: Control sample (basic vocoder reconstruction) [Audio1_Japanese_Control.wav]
- **Audio S1.2**: Depression-like modification [Audio1_Japanese_DepressionLike.wav]
- **Audio S1.3**: Mania-like modification [Audio1_Japanese_ManiaLike.wav]

To examine cross-linguistic generalizability, we also included modified English-language samples:

- **Audio S1.4**: English control sample [Audio2_English_Control.wav]
- **Audio S1.5**: English depression-like modification [Audio2_English_DepressionLike.wav]
- **Audio S1.6**: English mania-like modification [Audio2_English_ManiaLike.wav]

*Depression-Like Modifications*

The depression-modified samples exhibited significant reductions in MFCC variability indices, as shown in Figure S1.1. In particular, variability indices for MFCC 8 and MFCC 9 decreased notably, while the mean value of MFCC 4 increased. These changes are consistent with the correlations observed between these features and the diagnostic probability of depressive states in our primary analysis.

*Mania-Like Modifications*

Figure S1.2 presents the three acoustic features with the most significant changes between control and mania-modified samples, including one MFCC feature (MFCC dimension 5) and two spectral flux features. For mania-like modifications, the spectral flux variability indices, which are associated with speech clarity, increased significantly, as shown in Figure S1.2. Although spectral flux variability increased in the manipulated samples as intended, the direction of change for some features partially diverged from the correlations observed in the MS/MX diagnostic model, likely reflecting the complex and multidimensional nature of vocal expression in manic states.

*Section 9. Age and Sex Matching Results*

Age- and sex-matched undersampling was applied within each outer training fold; models were always evaluated on all participants of the held-out test fold, so matching affected only the composition of the training subsamples. Sex was matched exactly within pairs, and controls were selected within an age caliper that widened stepwise only when no closer match was available. Before matching, patients in the analysis cohorts were on average younger than HC (BP vs. HC: −3.4 years; DS vs. HC: −6.7 years; ES vs. HC: −2.6 years), whereas MS/MX patients were older than HC (+2.5 years). After matching, the residual mean age difference (patient group minus HC) across the matched training subsamples was +0.17 ± 0.27 years for BP vs. HC (100 subsamples), +0.16 ± 0.53 years for DS vs. HC, +0.29 ± 0.44 years for MS/MX vs. HC, and +0.14 ± 0.30 years for ES vs. HC (50 subsamples each); in every comparison with HC, at least 92% of subsamples showed a mean absolute age difference of 1 year or less, and none exceeded 1.5 years. For the inter-state comparisons within BP, the same matching procedure was applied, but the smaller participant pools limited the achievable balance: residual differences after matching were −0.05 ± 2.10 years for DS vs. ES, +4.23 ± 3.73 years for MS/MX vs. ES, and −5.00 ± 2.70 years for DS vs. MS/MX (cohort differences before matching: −5.8, +5.8, and −12.0 years, respectively). The residual age imbalance in the two inter-state comparisons involving MS/MX should be borne in mind when interpreting those results, although neither of these comparisons exceeded chance-level classification.

*Section 10. Feature-Selection Stability*

To evaluate the stability of feature selection and the associated risk of overfitting, we quantified, across all resampling iterations of the reported (headline) models (100 resamples for BP vs. HC [10 folds x 10 subsamplings] and 50 for each mood-state comparison [5 x 10]), the distribution of the number of selected features, the per-feature selection frequency, the coefficient-sign consistency, and the pairwise overlap (Jaccard index) of the selected feature sets, together with a chance-corrected stability index (Nogueira index)[15]. Results are shown in Table S10.

*Section 11. Medication Sensitivity Analysis*

Because all patients with bipolar disorder were medicated and no unmedicated bipolar group was available, medication confounding was examined within patients (healthy controls excluded; analyzed n = 60 for BP vs. HC, 32 for DS vs. HC, 20 for MS/MX vs. HC, 26 for ES vs. HC). One patient was excluded from all classification analyses because age, which is required for the age- and sex-matched undersampling, was unavailable; hence n = 60 rather than 61 for the comparison with healthy controls. For each patient, medication was coded from clinical records as the presence or absence of each class (antipsychotics [AP], mood stabilizers [MS], antidepressants [AD], anxiolytics/hypnotics [BZD]). We tested (i) whether the combined-arm diagnostic probability differed by medication class (Cohen d, permutation p), (ii) whether the task primary acoustic marker differed by class (Cohen d, permutation p, Benjamini-Hochberg FDR across the four classes)[16], (iii) the correlation between total medication burden and the diagnostic probability and marker (Spearman), and (iv) the same correlation after adjustment for symptom severity (partial Spearman). Acoustic-arm results were essentially identical to the combined arm.

Dose-response was additionally assessed in an exploratory manner using chlorpromazine equivalents (antipsychotics) and diazepam equivalents (anxiolytics and hypnotics)[17]. Daily doses had to be parsed from free-text prescription records, and entries such as as-needed orders and liquid formulations could not be quantified, so a daily dose was reliably extracted for only about 30% of antipsychotic and about 37% of anxiolytic or hypnotic prescriptions. Because the resulting equivalent burden is likely underestimated, dose-response results were treated as exploratory. Coding of medication class presence or absence and of the number of classes does not depend on dose parsing and is therefore unaffected. Mood stabilizers were analyzed as presence or absence only, because no validated cross-drug dose equivalent exists. All significance tests used permutation resampling with 2000 iterations. Because these tests resample already computed statistics rather than retraining the classifier, we used a count conventional for permutation inference (1000 to 10000), which keeps the Monte Carlo standard error of the p value small (about .005 at p near .05) and allows small p values to be estimated and ranked precisely. The severity covariate was Hamilton Depression Rating Scale-17 (HAMD-17) in all four models. Results are shown in Tables S11a, S11b, and S11c.

*Section 12. Modality-Specific Classification (BP vs. HC)*

This section reports modality-specific classification analyses. The primary BP vs. HC comparison was repeated with an otherwise identical pipeline using linguistic features only, acoustic features only, and both modalities combined (Figure S2).

*Section 13. Modality Composition of Significant Features*

The modality composition of the significant features is summarized in Table S12, and the significant linguistic features are listed in Table S13.

*Section 14. Operating-Point Metrics at a Fixed Threshold of 0.5*

To complement the Youden-index-based operating points reported in Table 4 of the main text, Table S14 reports the sensitivity, specificity, and accuracy of the four comparisons with HC at a fixed, pre-specified classification threshold of 0.5, computed from the participant-level averaged out-of-fold probabilities. In a robustness check, the mood-state versus HC classifications were repeated with the excluded participants included under a single harmonized inclusion rule; the AUC changed by no more than 0.02 in any comparison.

*Section 15. Temporal Structure of Data Collection*

This section summarizes the temporal structure of data collection in the analysis cohort (61 patients with BP and 99 HC), in which one recording session corresponds to one assessment visit. Table S15 reports the number of recording sessions per participant, the intervals between consecutive sessions, and the follow-up duration in each group.

Session-level mood-state labels follow the thresholds used in the classification analyses (DS: HAMD-17 ≥ 8, YMRS ≤ 7; MS/MX: YMRS ≥ 8; ES: HAMD-17 ≤ 7, YMRS ≤ 7). Of the 61 patients with BP, 42 were recorded in a single mood state across all of their sessions, whereas 19 were recorded in two or more states; patients observed in more than one state contribute to more than one state group in the comparisons with HC and were excluded from both groups in the direct inter-state comparisons. Among 116 consecutive state-labeled session pairs, 37 involved a change of mood state, most frequently from the depressive to the euthymic state (11 pairs) and from the euthymic to the depressive state (7 pairs).

**Supplementary References**

1.	Devlin J, Chang M-W, Lee K, Toutanova K. BERT: Pre-training of deep bidirectional Transformers for language understanding. arXiv [csCL]. 2018. Available from: http://arxiv.org/abs/1810.04805 [accessed Feb 15, 2025]

2.	Tohoku NLP Group. tohoku-nlp/bert-base-japanese-v3. Hugging Face. 2023. Available from: https://huggingface.co/tohoku-nlp/bert-base-japanese-v3

3.	Conneau A, Khandelwal K, Goyal N, Chaudhary V, Wenzek G, Guzmán F, Grave E, Ott M, Zettlemoyer L, Stoyanov V. Unsupervised cross-lingual representation learning at scale. Proceedings of the 58th Annual Meeting of the Association for Computational Linguistics Stroudsburg, PA, USA: Association for Computational Linguistics; 2020. p. 8440–8451.

4.	Kajiwara T, Chu C, Takemura N, Nakashima Y, Nagahara H. WRIME: A New Dataset for Emotional Intensity Estimation with Subjective and Objective Annotations. Proceedings of the 2021 Conference of the North American Chapter of the Association for Computational Linguistics: Human Language Technologies Online: Association for Computational Linguistics; 2021. p. 2095–2104.

5.	Kajiwara T. WRIME: A Japanese dataset for emotional intensity estimation with subjective and objective annotations. J Nat Lang Process Association for Natural Language Processing; 2021;28(3):907–912.

6.	Plutchik R. A General Psychoevolutionary Theory of Emotion. Theories of Emotion Elsevier; 1980. p. 3–33. ISBN:9780125587013

7.	Matsuda H. GiNZA - Practical Japanese NLP based on Universal Dependencies. J Nat Lang Process Association for Natural Language Processing; 2020 Sept 15;27(3):695–701.

8.	Eyben F, Wöllmer M, Schuller B. openSMILE: the Munich versatile and fast open-source audio feature extractor. Proceedings of the 18th ACM international conference on Multimedia New York, NY, USA: ACM; 2010. p. 1459–1462.

9.	Schuller B, Steidl S, Batliner A, Vinciarelli A, Scherer K, Ringeval F, Chetouani M, Weninger F, Eyben F, Marchi E, Mortillaro M, Salamin H, Polychroniou A, Valente F, Kim S. The INTERSPEECH 2013 computational paralinguistics challenge: social signals, conflict, emotion, autism. Interspeech 2013 ISCA; 2013. doi: 10.21437/interspeech.2013-56

10.	Morise M, Yokomori F, Ozawa K. WORLD: A vocoder-based high-quality speech synthesis system for real-time applications. IEICE Trans Inf Syst Institute of Electronics, Information and Communications Engineers (IEICE); 2016 July 1;E99.D(7):1877–1884.

11. Kominek J, Black AW. The CMU Arctic speech databases. 5th ISCA Workshop on Speech Synthesis (SSW 5) 2004. p. 223–224.

12.	Yamashita A, Sakai Y, Yamada T, Yahata N, Kunimatsu A, Okada N, Itahashi T, Hashimoto R, Mizuta H, Ichikawa N, Takamura M, Okada G, Yamagata H, Harada K, Matsuo K, Tanaka SC, Kawato M, Kasai K, Kato N, Takahashi H, Okamoto Y, Yamashita O, Imamizu H. Generalizable brain network markers of major depressive disorder across multiple imaging sites. PLoS Biol 2020 Dec;18(12):e3000966. PMID:33284797

13.	Hastie T, Tibshirani R, Wainwright M. Statistical learning with sparsity. Philadelphia, PA: Chapman & Hall/CRC; 2020. ISBN:9781138044975

14.	Westfall PH, Stanley Young S. Resampling-based multiple testing examples and methods for p-value adjustment. New York, NY: John Wiley & Sons; 1993. ISBN:9780471557616

15.	Nogueira S, Sechidis K, Brown G. On the stability of feature selection algorithms. J Mach Learn Res 2017;18(174):1–54.

16.	Benjamini Y, Hochberg Y. Controlling the false discovery rate: A practical and powerful approach to multiple testing. J R Stat Soc Series B Stat Methodol Oxford University Press (OUP); 1995 Jan 1;57(1):289–300.

17.	Inada T, Inagaki A. Psychotropic dose equivalence in Japan. Psychiatry Clin Neurosci Wiley; 2015 Aug;69(8):440–447. PMID:25601291

18.	Lakens D. Equivalence tests: A practical primer for t tests, correlations, and meta-analyses. Soc Psychol Personal Sci SAGE Publications; 2017 May;8(4):355–362. PMID:28736600

**Tables**

| Task | Modality | Features/resample, mean (SD) [range] | Rounds selecting 0 features | Features in ≥50% of resamples | Core sign consistency | Mean pairwise Jaccard | Nogueira index |
| --- | --- | --- | --- | --- | --- | --- | --- |
| BP vs. HC | Linguistic | 7 (10) [0-49] | 15/100 | 2 | 1.00 | 0.25 | 0.26 |
| BP vs. HC | Acoustic | 162 (112) [87-802] | 0/100 | 114 | 1.00 | 0.49 | 0.57 |
| BP vs. HC | Combined | 168 (119) [90-825] | 0/100 | 116 | 1.00 | 0.48 | 0.56 |
| DS vs. HC | Linguistic | 8 (7) [0-28] | 11/50 | 3 | 1.00 | 0.24 | 0.31 |
| DS vs. HC | Acoustic | 190 (234) [0-1207] | 5/50 | 76 | 1.00 | 0.20 | 0.29 |
| DS vs. HC | Combined | 214 (225) [0-1003] | 2/50 | 97 | 1.00 | 0.22 | 0.32 |
| MS/MX vs. HC | Linguistic | 40 (37) [0-126] | 10/50 | 18 | 0.998 | 0.22 | 0.21 |
| MS/MX vs. HC | Acoustic | 337 (488) [0-2004] | 3/50 | 88 | 1.00 | 0.18 | 0.22 |
| MS/MX vs. HC | Combined | 364 (613) [0-2542] | 2/50 | 74 | 1.00 | 0.18 | 0.18 |
| ES vs. HC | Linguistic | 24 (27) [0-115] | 13/50 | 6 | 1.00 | 0.20 | 0.19 |
| ES vs. HC | Acoustic | 151 (92) [40-416] | 0/50 | 97 | 1.00 | 0.29 | 0.42 |
| ES vs. HC | Combined | 154 (94) [40-445] | 0/50 | 97 | 1.00 | 0.29 | 0.42 |

Table S10. Feature-selection stability across resampling iterations. Sign consistency is the mean over the reproducible core (features selected in at least 50% of resamples); the median per-feature sign consistency across all selected features was 1.00 in every model arm. Rounds selecting zero features are concentrated in the linguistic and mood-state arms and are included in the Jaccard and Nogueira computations. Set-level overlap was moderate for BP vs. HC and low for the smaller mood-state comparisons.

| Task | Medication class | n (on/off) | Diagnostic prob., Cohen d | Diagnostic prob., p | Primary marker, Cohen d | Primary marker, p |
| --- | --- | --- | --- | --- | --- | --- |
| BP vs. HC | Antipsychotics | 44/16 | +0.41 | .160 | -0.42 | .143 |
| BP vs. HC | Mood stabilizers | 46/14 | +0.02 | .959 | +0.22 | .472 |
| BP vs. HC | Antidepressants | 20/40 | +0.10 | .709 | +0.22 | .425 |
| BP vs. HC | Anxiolytics/hypnotics | 29/31 | -0.10 | .690 | +0.14 | .591 |
| DS vs. HC | Antipsychotics | 25/7 | -0.42 | .331 | +0.39 | .373 |
| DS vs. HC | Mood stabilizers | 22/10 | +0.22 | .578 | -0.60 | .135 |
| DS vs. HC | Antidepressants | 14/18 | +0.34 | .341 | +0.48 | .190 |
| DS vs. HC | Anxiolytics/hypnotics | 16/16 | -0.02 | .972 | -0.27 | .454 |
| MS/MX vs. HC | Antipsychotics | 16/4 | -0.72 | .208 | -0.44 | .429 |
| MS/MX vs. HC | Mood stabilizers | 16/4 | -0.10 | .863 | -0.32 | .550 |
| MS/MX vs. HC | Antidepressants | 6/14 | -0.56 | .283 | +0.17 | .745 |
| MS/MX vs. HC | Anxiolytics/hypnotics | 12/8 | -0.81 | .091 | +0.14 | .780 |
| ES vs. HC | Antipsychotics | 14/12 | +1.17 | .005 | -0.78 | .046 |
| ES vs. HC | Mood stabilizers | 21/5 | -0.32 | .532 | +0.03 | .966 |
| ES vs. HC | Antidepressants | 9/17 | -0.10 | .820 | +0.16 | .715 |
| ES vs. HC | Anxiolytics/hypnotics | 9/17 | -0.23 | .611 | -0.48 | .278 |

Table S11a. Medication sensitivity analysis: diagnostic probability and primary acoustic marker by medication class (within patients; combined arm). Values are Cohen d (medication present vs. absent) with permutation p. Marker class p values were Benjamini-Hochberg FDR corrected across the four classes; no class was significant after correction in any task (the largest, ES antipsychotics for the marker, was nominal p = 0.046, q > 0.05). Lithium was additionally tested as an exploratory single-drug marker contrast (underpowered): BP d = -0.36, p = 0.169; DS d = +0.59, p = 0.151; MS/MX d = -0.82, p = 0.074; ES d = +0.26, p = 0.588.

| Task | Load vs. diagnostic prob. (ρ, p) | Load vs. marker (ρ, p) | Severity-adjusted load vs. marker (ρ, p) | Marker class tests significant after FDR |
| --- | --- | --- | --- | --- |
| BP vs. HC | 0.08, .574 | -0.11, .407 | -0.10, .419 | 0 / 4 |
| DS vs. HC | 0.08, .654 | -0.10, .563 | -0.01, .960 | 0 / 4 |
| MS/MX vs. HC | -0.42, .067 | 0.15, .542 | 0.12, .618 | 0 / 4 |
| ES vs. HC | 0.21, .271 | -0.46, .023 | -0.45, .017 | 0 / 4 |

Table S11b. Medication burden (number of classes) vs. diagnostic probability and marker (Spearman), and the marker association after adjustment for symptom severity (partial Spearman). Dose-response using chlorpromazine and diazepam equivalents was exploratory only, because a daily dose could be reliably parsed for about 30% of antipsychotic and about 37% of anxiolytic/hypnotic records. One exception to the otherwise null pattern: in ES vs. HC the diagnostic probability was higher in antipsychotic users (d = +1.17, p = 0.005) and the marker correlated with total medication burden even after severity adjustment (ρ = -0.45, p = 0.017), indicating that the euthymic-state findings cannot be fully separated from medication (see Limitations).

| Model | Medication class | n users/non-users | Cohen d | 90% CI | P | q | Equivalence (0.5 SD) |
| --- | --- | --- | --- | --- | --- | --- | --- |
| BP vs. HC | Antipsychotics | 44/16 | -0.42 | -1.06 to +0.09 | .143 | .574 | Not demonstrated |
| BP vs. HC | Mood stabilizers | 46/14 | +0.22 | -0.19 to +0.60 | .472 | .591 | Not demonstrated |
| BP vs. HC | Antidepressants | 20/40 | +0.22 | -0.32 to +0.71 | .425 | .591 | Not demonstrated |
| BP vs. HC | Anxiolytics/hypnotics | 29/31 | +0.14 | -0.30 to +0.54 | .591 | .591 | Not demonstrated |
| BP vs. HC | Lithium (exploratory) | 28/32 | -0.36 | -0.77 to +0.06 | .169 | n/a | Not demonstrated |
| DS vs. HC | Antipsychotics | 25/7 | +0.39 | -0.16 to +1.13 | .373 | .454 | Not demonstrated |
| DS vs. HC | Mood stabilizers | 22/10 | -0.60 | -1.17 to -0.10 | .135 | .381 | Not demonstrated |
| DS vs. HC | Antidepressants | 14/18 | +0.48 | -0.11 to +1.18 | .190 | .381 | Not demonstrated |
| DS vs. HC | Anxiolytics/hypnotics | 16/16 | -0.27 | -0.91 to +0.33 | .454 | .454 | Not demonstrated |
| DS vs. HC | Lithium (exploratory) | 9/23 | +0.59 | +0.04 to +1.25 | .151 | n/a | Not demonstrated |
| MS/MX vs. HC | Antipsychotics | 16/4 | -0.44 | -1.05 to +0.22 | .429 | .780 | Not demonstrated |
| MS/MX vs. HC | Mood stabilizers | 16/4 | -0.32 | -1.34 to +0.90 | .550 | .780 | Not demonstrated |
| MS/MX vs. HC | Antidepressants | 6/14 | +0.17 | -0.78 to +2.16 | .745 | .780 | Not demonstrated |
| MS/MX vs. HC | Anxiolytics/hypnotics | 12/8 | +0.14 | -0.50 to +1.21 | .780 | .780 | Not demonstrated |
| MS/MX vs. HC | Lithium (exploratory) | 11/9 | -0.82 | -1.66 to -0.21 | .074 | n/a | Not demonstrated |
| ES vs. HC | Antipsychotics | 14/12 | -0.78 | -1.33 to -0.24 | .046 | .186 | Not demonstrated |
| ES vs. HC | Mood stabilizers | 21/5 | +0.03 | -0.87 to +0.53 | .966 | .966 | Not demonstrated |
| ES vs. HC | Antidepressants | 9/17 | +0.16 | -0.87 to +0.81 | .715 | .954 | Not demonstrated |
| ES vs. HC | Anxiolytics/hypnotics | 9/17 | -0.48 | -0.90 to -0.01 | .278 | .556 | Not demonstrated |
| ES vs. HC | Lithium (exploratory) | 13/13 | +0.26 | -0.47 to +0.76 | .588 | n/a | Not demonstrated |

Table S11c. Detailed results for the primary acoustic marker by medication class (within patients). These tests use the acoustic marker and the medication record only, so the values are identical in the acoustic and combined arms. Cohen d is the standardized difference between users and non-users, with a bootstrap 90% confidence interval (1000 resamples). p values are permutation p values (2000 resamples), and q values are Benjamini-Hochberg adjusted across the four confirmatory medication classes within each model[16]. Equivalence was assessed by two one-sided tests against a margin of 0.5 SD; not demonstrated indicates that the confidence interval crossed the margin, which is a failure to demonstrate equivalence rather than evidence of an effect[18]. Lithium was examined as an exploratory single-agent contrast and was not included in the false discovery rate family. No medication class was significant after correction in any model (0 of 4 in each). Primary markers: BP vs. HC, pcm_fftMag_spectralFlux_sma_peakRangeAbs; DS vs. HC, voicingFinalUnclipped_sma_lpc1; MS/MX vs. HC, pcm_fftMag_psySharpness_sma_de_lpc1; ES vs. HC, pcm_fftMag_spectralFlux_sma_meanRisingSlope.

| Comparison | Significant features (n) | Acoustic (n) | Linguistic (n) | Acoustic (%) |
| --- | --- | --- | --- | --- |
| BP vs. HC | 178 | 174 | 4 | 97.8 |
| DS vs. HC | 121 | 118 | 3 | 97.5 |
| MS/MX vs. HC | 108 | 104 | 4 | 96.3 |
| ES vs. HC | 125 | 123 | 2 | 98.4 |

Table S12. Modality composition of the statistically significant features (permutation-based selection, family-wise error corrected) for each comparison.

| Feature (Japanese) | English gloss | BP | DS | MS/MX | ES | n |
| --- | --- | --- | --- | --- | --- | --- |
| particles_to_ratio | ratio of the case particle *to* | ✓ |  | ✓ | ✓ | 3 |
| sadness_Variability | variability of sadness sentiment | ✓ | ✓ |  |  | 2 |
| particles_kara_ratio | ratio of the particle *kara* | ✓ | ✓ |  |  | 2 |
| ratio_助詞-格助詞 | ratio of case-particle POS |  |  | ✓ | ✓ | 2 |
| num_感動詞-フィラー | count of filler interjections |  |  | ✓ |  | 1 |
| fear_Variability | variability of fear sentiment |  | ✓ |  |  | 1 |
| ratio_動詞-一般 | ratio of general verbs | ✓ |  |  |  | 1 |
| particles_wo_ratio | ratio of the case particle *wo* |  |  | ✓ |  | 1 |

Table S13. Statistically significant linguistic features across the four comparisons with healthy controls (Japanese feature names shown; n is the number of comparisons in which the feature was significant).

| Comparison | Sensitivity | Specificity | Accuracy |
| --- | --- | --- | --- |
| BP vs. HC (60 vs. 99) | 0.783 (47/60) | 0.879 (87/99) | 0.843 (134/159) |
| DS vs. HC (32 vs. 97) | 0.750 (24/32) | 0.814 (79/97) | 0.798 (103/129) |
| MS/MX vs. HC (20 vs. 97) | 0.800 (16/20) | 0.835 (81/97) | 0.829 (97/117) |
| ES vs. HC (26 vs. 97) | 0.731 (19/26) | 0.835 (81/97) | 0.813 (100/123) |

Table S14. Operating-point metrics for the comparisons with HC at a fixed, pre-specified threshold of 0.5, computed from the participant-level averaged out-of-fold probabilities. These complement the Youden-index-based values in Table 4 of the main text, whose cut-point is selected on the evaluation data and may therefore be optimistic.

| Metric | BP (n=61) | HC (n=99) |
| --- | --- | --- |
| Total recording sessions | 180 | 205 |
| Sessions per participant, mean (SD) | 2.95 (1.64) | 2.07 (0.82) |
| Sessions per participant, median | 3 | 2 |
| Sessions per participant, range | 1-5 | 1-5 |
| Participants with 1 session | 17 | 20 |
| Participants with 2 sessions | 13 | 61 |
| Participants with 3 sessions | 5 | 10 |
| Participants with 4 sessions | 8 | 7 |
| Participants with 5 sessions | 18 | 1 |
| Consecutive-session intervals, n | 116 | 104 |
| Interval, median (IQR), days | 51 (35-91) | 35 (28-49) |
| Interval, range, days | 21-882 | 18-439 |
| Intervals shorter than 30 days, n | 23 | 37 |
| Participants with ≥2 dated sessions | 44 | 78 |
| Follow-up, median (IQR), days | 154 (93-329) | 35 (28-96) |
| Follow-up, range, days | 28-1127 | 18-596 |
| Follow-up, median (max), months | 5.1 (37.0) | 1.1 (19.6) |

Table S15. Temporal structure of data collection in the analysis cohort. One recording session corresponds to one assessment visit. Intervals were computed between consecutive dated sessions; follow-up is the interval from the first to the last session and is defined for participants with at least two dated sessions. Session dates were available for 380 of 385 sessions. The study protocol specified two to five assessment visits at intervals of approximately one month; the table reports the distribution actually observed. BP, bipolar disorder; HC, healthy controls; SD, standard deviation; IQR, interquartile range.

**Figure legends**

**Figure S1.1. Depression-like modifications compared to control samples. Box plots illustrating significant differences in MFCC features between control (gray) and depression-modified (blue) samples. Left: Arithmetic mean of MFCC dimension 4 shows a significant increase (*p* < 0.001, r = 1.000). Center: Standard deviation rising slope of MFCC dimension 8 shows a significant decrease (*p* = 0.003, r = −0.778). Right: Standard deviation falling slope of MFCC dimension 9 shows a significant decrease (*p* < 0.001, r = −0.948).**

**Figure S1.2. Mania-like modifications compared to control samples.** Box plots illustrating significant differences in spectral flux features between control (gray) and mania-modified (orange) samples. Left: MFCC dimension 5 (second quartile) shows a significant decrease (*p* < 0.001, r = −0.987). Center: Mean rising slope of pitch-corrected magnitude spectral flux (smoothed) shows a significant increase (*p* < 0.001, r = 0.961). Right: Range of pitch-corrected magnitude spectral flux (smoothed) shows a significant increase (*p* < 0.001, r = 0.961).

Figure S2. Modality-specific classification of BP vs. HC. Kernel density estimates of the per-participant diagnostic probability for healthy controls (gray) and patients with bipolar disorder (red), for models trained on (a) linguistic features only (211 candidate features), (b) acoustic features only (3,473 candidate features), and (c) both modalities combined (3,684 candidate features). Candidate counts are the features available to each model after the 2,900 volume-dependent acoustic features were excluded from the 6,373 extracted acoustic features before model training, within each cross-validation fold (Section 4). The dashed line marks the 0.5 classification threshold. The acoustic-only and combined models yielded near-identical distributions and AUCs (0.910 vs. 0.911), whereas the linguistic-only model showed substantial overlap (AUC 0.651), indicating that discrimination was driven by acoustic features with negligible incremental contribution from linguistic features. AUCs are from the seed-fixed re-analysis and were stable across five random seeds (SD ≤ 0.01).

**Figures**

Figure S1.1.

Figure S1.2.

Figure S2.
